# Toward novel treatment against filariasis: Insight into genome-wide co-evolutionary analysis of filarial nematodes and *Wolbachia*

**DOI:** 10.3389/fmicb.2023.1052352

**Published:** 2023-03-22

**Authors:** Arporn Wangwiwatsin, Siriyakorn Kulwong, Jutarop Phetcharaburanin, Nisana Namwat, Poramate Klanrit, Watcharin Loilome, Wanchai Maleewong, Adam J. Reid

**Affiliations:** ^1^Department of Biochemistry, Faculty of Medicine, Khon Kaen University, Khon Kaen, Thailand; ^2^Cholangiocarcinoma Research Institute, Khon Kaen University, Khon Kaen, Thailand; ^3^Khon Kaen University Phenome Centre, Khon Kaen University, Khon Kaen, Thailand; ^4^Department of Parasitology, Faculty of Medicine, Khon Kaen University, Khon Kaen, Thailand; ^5^Parasite Genomics Group, Wellcome Sanger Institute, Hinxton, United Kingdom; ^6^The Gurdon Institute, University of Cambridge, Cambridge, United Kingdom

**Keywords:** filarial nematode, *Wolbachia*, co-evolution, mirrortree, protein–protein interactions, genomics

## Abstract

Infectious diseases caused by filarial nematodes are major health problems for humans and animals globally. Current treatment using anti-helminthic drugs requires a long treatment period and is only effective against the microfilarial stage. Most species of filarial nematodes harbor a specific strain of *Wolbachia* bacteria, which are essential for the survival, development, and reproduction of the nematodes. This parasite-bacteria obligate symbiosis offers a new angle for the cure of filariasis. In this study, we utilized publicly available genome data and putative protein sequences from seven filarial nematode species and their symbiotic *Wolbachia* to screen for protein–protein interactions that could be a novel target against multiple filarial nematode species. Genome-wide *in silico* screening was performed to predict molecular interactions based on co-evolutionary signals. We identified over 8,000 pairs of gene families that show evidence of co-evolution based on high correlation score and low false discovery rate (FDR) between gene families and obtained a candidate list that may be keys in filarial nematode–*Wolbachia* interactions. Functional analysis was conducted on these top-scoring pairs, revealing biological processes related to various signaling processes, adult lifespan, developmental control, lipid and nucleotide metabolism, and RNA modification. Furthermore, network analysis of the top-scoring genes with multiple co-evolving pairs suggests candidate genes in both *Wolbachia* and the nematode that may play crucial roles at the center of multi-gene networks. A number of the top-scoring genes matched well to known drug targets, suggesting a promising drug-repurposing strategy that could be applicable against multiple filarial nematode species.

## 1. Introduction

Filarial nematodes comprise multiple species and they are a major health problem globally. These parasites cause diseases such as lymphatic filariasis (elephantiasis), onchocerciasis (river blindness), and dirofilariasis (heartworm disease), resulting in disfigurement, social stigma, and, in some cases, permanent disability. These diseases affect up to 50 million people worldwide, and over 1.5 billion people are at risk of infection. Some species also cause diseases in animals, making filariasis a problem for both human and veterinary medicine (Vos et al., 2016; Lustigman et al., 2017). The life cycle of the filarial nematode involves biting insect vectors and mammalian hosts. In the mammalian host, the larval stages reside in regional lymphatic vessels or nodules and develop into mating adults, which release microfilaria into the circulation system (Cross, 2011).

The majority of filarial nematodes co-exist with an obligatory endosymbiotic bacterium called *Wolbachia*, a gram-negative alphaproteobacterium in the order Rickettsia. *Wolbachia* is present in all life stages of the nematode, with a marked increase once the nematode passes from insect vector to mammalian host, and another sharp increase as the nematodes develop into adults (Fenn and Blaxter, 2004; McGarry et al., 2004). The presence of *Wolbachia* is essential for worm survival, development, embryogenesis, and reproduction (Scott et al., 2012; Taylor et al., 2013). At the molecular level, *Wolbachia*–filarial nematode interactions are associated with haem metabolism, nucleotide metabolism, fatty acid synthesis, as well as folic acid synthesis (Slatko et al., 2010; Taylor et al., 2013). When the phylogenetic trees of the nematode and the *Wolbachia* are compared, they closely reflect one another, suggesting co-evolution between the nematode and the Wolbachia – a phenomenon often found in organisms with a long-term, tightly associated relationship (Bandi et al., 1999; Casiraghi et al., 2001; Ferri et al., 2011). The elimination of filarial nematode infection might be achieved through disruption of this essential symbiotic relationship, either by targeting the bacterium or targeting the molecular interactions between the worm and the bacterium. The latter approach is potentially more specific for filariasis treatment and may reduce the risk of antibiotic resistance.

Current treatment with anthelminthic drugs such as ivermectin is sub-optimal against the adult stage (Basáñez et al., 2008; Diawara et al., 2009; Tamarozzi et al., 2011). Signs of drug resistance have been observed (Schwab et al., 2006; Laman et al., 2022); hence, alternative treatments are urgently needed. New treatment trials have focused on killing the *Wolbachia* endosymbiont as well as the nematode by using a combination of antibiotics and anthelminthic drugs (Taylor et al., 2014; Turner et al., 2017). Treating experimentally infected animal models with antibiotics affected the reproduction capacity of the nematodes, as indicated by the reduced number of microfilaria, and eventually the killing of the adult stage (Bandi et al., 1999; Volkmann et al., 2003; Aljayyoussi et al., 2017; Specht et al., 2018). However, mass-drug administration of antibiotics disrupts the microbiota of the treated individual as well as risks the eventual development of antibiotic-resistant bacteria. Screenings for other anti-*Wolbachia* drugs, as well as attempting to target nematode–*Wolbachia* interactions, are underway (Landmann et al., 2012; Scott et al., 2012; Poopandi et al., 2021). However, these attempts often focus on a single filarial nematode species. Recently, with the publicly available genomes for many filarial nematode species and their *Wolbachia*, we aim to reveal targets that could be relevant against multiple species.

Proteins that interact with one another tend to co-evolve to maintain their functions, and this allows researchers to use co-evolutionary signals to predict protein–protein interactions within a species, known as the *mirrortree* approach (Pazos and Valencia, 2001; Goh and Cohen, 2002). In this well-established approach, one can predict protein–protein interactions by comparing amino acid sequences in related species and screen for gene trees that are very similar to each other (Shoemaker and Panchenko, 2007; Pazos and Valencia, 2008; Ochoa et al., 2015). This approach has been tested with data from multiple species, both prokaryotic and eukaryotic genomes (Goh and Cohen, 2002; Mier et al., 2017). Over time, the methods have been further developed to improve their accuracy and utility (Ochoa and Pazos, 2014). In particular, some genes may appear to be co-evolving because of the phylogenetic signal between species. Therefore, the Tree-of-Life (tol) mirrortree approach subtracts the species trees from the gene tree prior to gene co-evolutionary analysis (Pazos et al., 2005). Furthermore, a *p*-value calculation that is designed for analyzing multiple phylogenetic trees can be applied to the analysis of each gene pair (Ochoa et al., 2015). The approach has revealed a signal of co-evolution between interacting gene products in the mitochondrial and nuclear genomes (Ochoa and Pazos, 2014), which suggest that the approach could be applicable to interactions that occur between different species (two sets of genomes).

Here, we used publicly available genome data of seven species of filarial nematodes and their *Wolbachia*, namely, *Brugia malayi*, *Brugia pahangi*, *Dirofilaria immitis*, *Wuchereria bancrofti*, *Litomosoides sigmodontis*, *Onchocerca volvulus*, and *Onchocerca ochengi*, and applied co-evolutionary screening at the protein sequence level (mirrortree approach) to predict molecular interactions between the filarial nematode and the *Wolbachia*. By integrating the orthologous genes in the nematode and *Wolbachia* across seven species, the outcome provided here is expected to be applicable against the range of filariasis-causing agents. The results are consistent with the known biology of the symbiotic relationship as well as provide pointers to specific molecules and pathways that can be shortlisted for further anti-filarial screening.

## 2. Materials and methods

### 2.1. Source of genomic data

Publicly available genomic data of filarial nematode and its symbiotic *Wolbachia* are obtained from multiple databases, as shown in Table 1. All annotated genes of each species were collected as FASTA format of amino acid sequences.

**Table 1:** Genome data used for analysis.

Organism	Nematode sequence source and version	Wolbachia sequence source and version
*Brugia malayi*	WormBase ParaSite: r13 (PRJNA10729)	Ensembl Bacteria: r44 (GCA_000008385)
*Brugia pahangi*	WormBase ParaSite: r13 (PRJEB497)	WormBase ParaSite: r13 (PRJEB497)
*Onchocerca ochengi*	WormBase ParaSite: r13 (PRJEB1204)	Ensembl Bacteria: r44 (GCA_000306885.1)
*Onchocerca volvulus*	WormBase ParaSite: r13 (PRJEB513)	RefSeq (GCF_000530755.1)
*Wuchereria bancrofti*	WormBase ParaSite: r13 (PRJNA275548)	RefSeq (GCF_002204235.2)
*Dirofilaria immitis*	WormBase ParaSite: r13 (PRJEB1797)	nematodes.org (wDi.2.2)
*Litomosoides sigmodontis*	WormBase ParaSite: r13 (PRJEB3075)	nematodes.org (wLs.2.0)

### 2.2. Annotation of *Brugia pahangi Wolbachia* genome

For *Wolbachia* of *B. pahangi*, the reference genome was available as a by-product of *B. pahangi* genome sequencing (International Helminth Genomes Consortium, 2019) but no annotation was provided. We, therefore, annotated this genome using prokka (version 1.13; Seemann, 2014) with default parameters and using its default core BLAST+ database, UniProtKB (SwissProt), for protein-coding genes.

### 2.3. Overview of the co-evolutionary screening workflow

**Figure 1:**
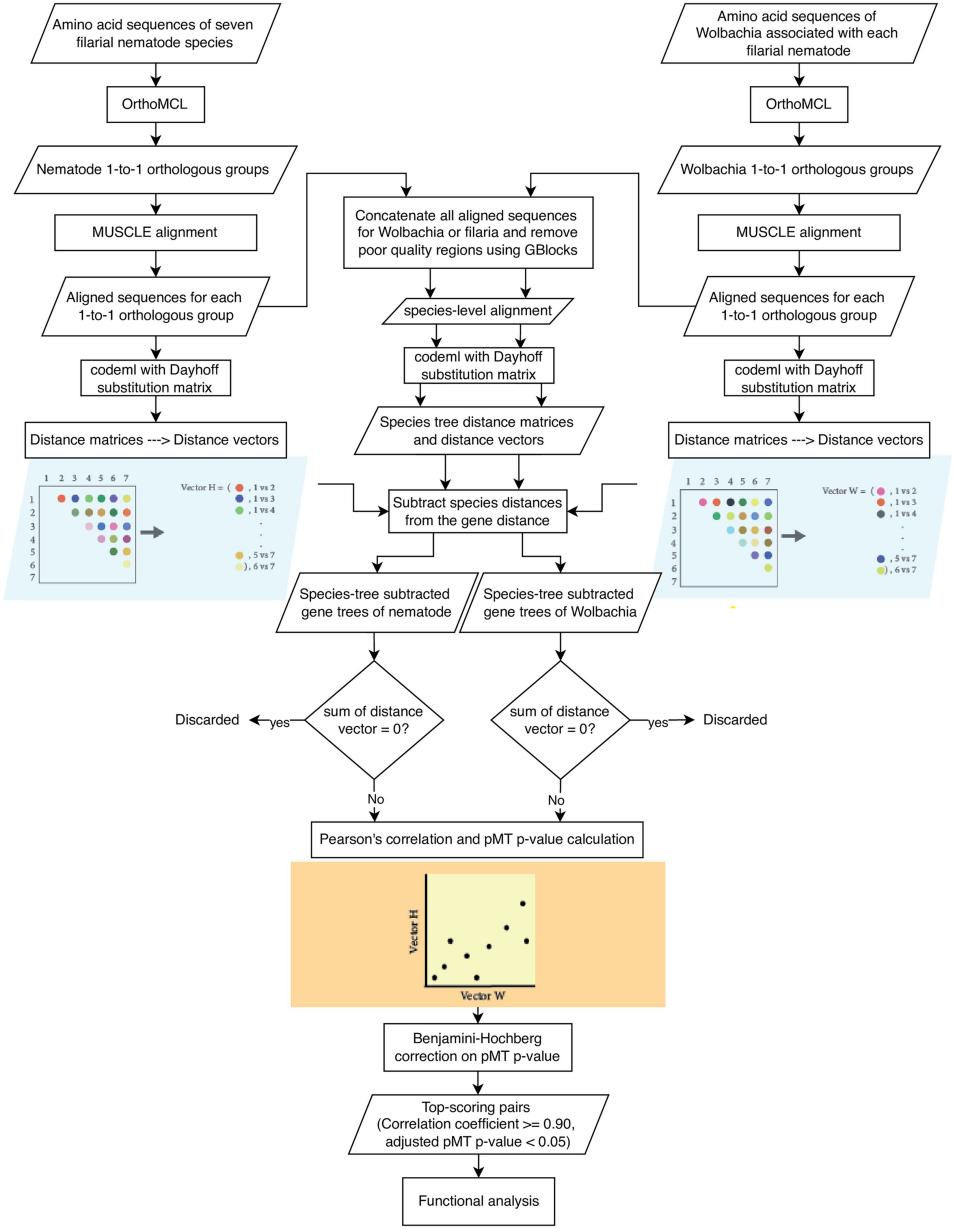
Analysis workflow. Rectangles indicate processes. Parallelograms indicate data. Diamonds indicate decisions and filtering. The conversion of a distance matrix to its distance vector, and the correlation analysis of distance vectors are shown here for only one matrix from nematode and *Wolbachia* each.

The workflow is presented in Figure 1. In brief, orthologous groups for the protein sequences of seven filarial nematode species and seven *Wolbachia* species were identified. Orthologous groups with one gene from each species (of either nematodes or bacteria) were kept for downstream analysis. Within each group, protein sequences were aligned using MUSCLE, and a distance matrix between species was created with codeml using the Dayhoff matrix. The species tree distance matrices of the nematodes or *Wolbachia* were created and subtracted from the gene distance matrices by taking away from each gene tree value the distance between corresponding species in the species tree distance matrix based on the tol-mirrortree method (Pazos et al., 2005). Then, the subtracted gene trees were used for correlation analysis. After the species-tree subtraction, the distance matrices from each 1-to-1 orthologous group of the nematode were then compared with the distance matrices of the *Wolbachia* in an all-against-all manner. The correlation coefficients between the nematode and *Wolbachia* matrices were used as an indicator of phylogenetic tree similarity, and hence the co-evolution signal between each pair of nematode–*Wolbachia* gene groups (Pazos and Valencia, 2001). Statistical significance of the correlation was assessed using the *p*-value calculation based on the method of pMT *p*-value Ochoa et al. (2015), followed by multiple-testing correction using the Benjamini–Hochberg method (Benjamini and Hochberg, 1995). Nematode–*Wolbachia* gene pairs with the top scores for both correlation coefficient and pMT *p*-value were taken for further functional analysis.

### 2.4. Identification of 1-to-1 ortholog

OrthoMCL (version 1.4; Li et al., 2003) was used to identify orthologous groups with one gene from each species (1-to-1 ortholog). For the nematode dataset, all protein sequences from all seven species were combined and used as a reference database for BLASTp (version 2.2.26; Altschul et al., 1990). BLASTp for each protein sequence was performed with -e 0.01. The BLASTp result and a table containing a list of gene IDs for each species were used as input for OrthoMCL run with mode 3. The output from OrthoMCL was filtered to keep only the orthologous groups with one gene from each nematode species. The same pipeline was applied to the *Wolbachia* dataset to obtain the *Wolbachia* 1-to-1 orthologous groups.

### 2.5. Gene sequence alignment and analysis of evolutionary distances

Within each 1-to-1 orthologous group, the protein sequences of its member were aligned using MUSCLE alignment (version 6.4.2; Edgar, 2004). The distance matrix between each protein sequence within a group was calculated with the Dayhoff matrix using *codeml* (version 4.9; Yang, 2007) with default parameters and runmode = −2. Each distance matrix was converted into a distance vector while maintaining the species order across all groups, and these vectors were then used for correlation analysis.

### 2.6. Removal of speciation background from gene evolutionary distances

Based on the method of Pazos et al. (2005), subtracting species trees can help remove the correlation that is a result of co-evolution at the species level. In this study, the species tree of the nematodes and the *Wolbachia* was generated by concatenating all of their aligned 1-to-1 orthologs, using a script concatenate_fasta_alignments.py available from Aunin et al. and their associated GitHub^1^ (Aunin et al., 2020). GBlocks (version 0.91b; Castresana, 2000) was us ed to remove alignment positions with poor quality (containing missing sequences from at least one species). Distance matrix and distance vectors were created in the same way as the gene orthologous groups. The species tree distance vector of the nematode was subtracted from each nematode gene distance vector, and the same process was performed for the *Wolbachia* species tree. The resulting gene distance vectors, with species tree subtracted, were then used for the calculation of Pearson’s correlation coefficient.

### 2.7. Correlation between nematode and *Wolbachia* genes

Each distance vector of the nematodes, with or without species tree subtraction, was correlated with all distance vectors of the *Wolbachia* in an all-against-all manner. Following the method developed by Pazos and Valencia (2001), the similarity of the distance vectors, i.e., the similarity of the phylogenetic trees, was measured by Pearson’s correlation coefficient. The *p*-value for each correlation was calculated using the pMT method of Ochoa et al. (2015), which has been designed specifically for the mirrortree approach. It accounts for the fact that each value between the distance vectors is not entirely independent of one another because they are limited by the species present in each tree. Therefore, when creating the background distribution for *p*-value calculation, the pMT method limits its randomization to only shuffle the data points of the same species between trees. By doing so, the resulting background distribution will be trees that each contain all species, and each species is present only once in a given tree – a more realistic situation for phylogenetic data. Each branch shuffling is counted as one iteration. For our analysis, with ~5,000 ortholog groups and 7 branches in each group, the pMT was run with 100,000 iterations. The pMT *p*-values were corrected for multiple testing using the Benjamini–Hochberg method.

### 2.8. Functional analysis of top-scoring results

To investigate the genes with the strong co-evolutionary signal between the nematodes and the *Wolbachia*, top-scoring genes (Pearson’s correlation coefficient ≥ 0.90, Benjamini–Hochberg adjusted pMT *p*-value < 0.05) between nematodes and *Wolbachia* with species tree subtraction were used for downstream analyses.

Genes from *B. malayi* (Bm) and *Wolbachia* associated with *B. malayi* (wBm) were used for downstream functional analyses due to its more complete genome among all filarial nematodes in this study. GO term annotations for *B. malayi* genes were obtained from WormBase ParaSite release 13 (Howe et al., 2017; Bolt et al., 2018) using the “BioMart” feature. GO term annotation of *Wolbachia* associated with *B. malayi* was obtained using InterProScan (version 5.55-88.0; Jones et al., 2014). The InterProScan was run using default parameters with -goterms -pa. GO term enrichment was performed using topGO (Alexa and Rahnenfuhrer, 2021) with a weight algorithm and Fisher’s exact test using all annotated genes of *B. malayi* or *Wolbachia* associated with *B. malayi* as the background dataset. GO term enrichment was performed separately for genes of *B. malayi* or *Wolbachia* associated with *B. malayi*.

Cytoscape (version 3.8.2; Shannon et al., 2003) was used for visualizing the network of co-evolving groups and for identifying groups that appeared to be centers of potential interactions. Only the connections linking nematode and *Wolbachia* genes were included in the network analysis and not the nematode-nematode or *Wolbachia*-*Wolbachia* connections. Nematode and *Wolbachia* nodes with a high number of connections (correlated with multiple gene groups from their symbiotic partner) were annotated as the top 5% and top 1% most highly connected nodes, which refer to being above 95% quartile and 99% quartile, respectively. These top 5% nodes and their first neighbors (nodes that were directly connected to them) were isolated from the full network and further investigated.

### 2.9. KEGG pathway mapping

The top-scoring genes of *B. malayi* and *Wolbachia* associated with *B. malayi* were mapped to KEGG ID using KEGG BlastKOALA (version 2.2; Kanehisa and Goto, 2000; Kanehisa et al., 2016) with amino acid sequences as query and searched against either *B. malayi* (taxonomy ID 6279) or *Wolbachia* of *B. malayi* (taxonomy ID 292805) reference. The resulting KEGG IDs were visualized on reference KEGG pathways using KEGG Mapper (version 5.0; Kanehisa et al., 2022). Green color denotes *Wolbachia* genes; yellow and red colors denote nematode genes. The denser colors represent the top 5% and top 1% most highly connected nodes of *Wolbachia* and nematode, respectively.

### 2.10. Druggability screening

The top 1% most highly connected nodes of the *Wolbachia* and nematode were screened for known drug targets using DrugBank online^2^ (version 5.1.9; Wishart et al., 2006) using the “target search” feature. The amino acid sequences of the *B. malayi* and *Wolbachia* associated with *B. malayi* were used as input. BLAST parameters were used as a default in the DrugBank online (cost to open a gap = −1, cost to extend a gap = −1, penalty for mismatch = −3, reward for match = 1, and expectation value (*E*-value) for reporting = 10^−5^, with low compositional complexity masking/filtering). Resulting target hits were further filtered for those with “approved” or “vet-approved” status.

## 3. Results

The seven nematode genomes used in this study had an average of 12,102 protein-coding genes (range 10,246–14,674), and the *Wolbachia* genomes had 845 protein-coding genes (range 647–1,119). We identified 4,506 orthologous groups of genes for the filarial nematodes and 539 groups for the *Wolbachia* (1-to-1 orthologs only).

### 3.1. A number of genes appeared to co-evolve between nematode and *Wolbachia*

**Figure 2:**
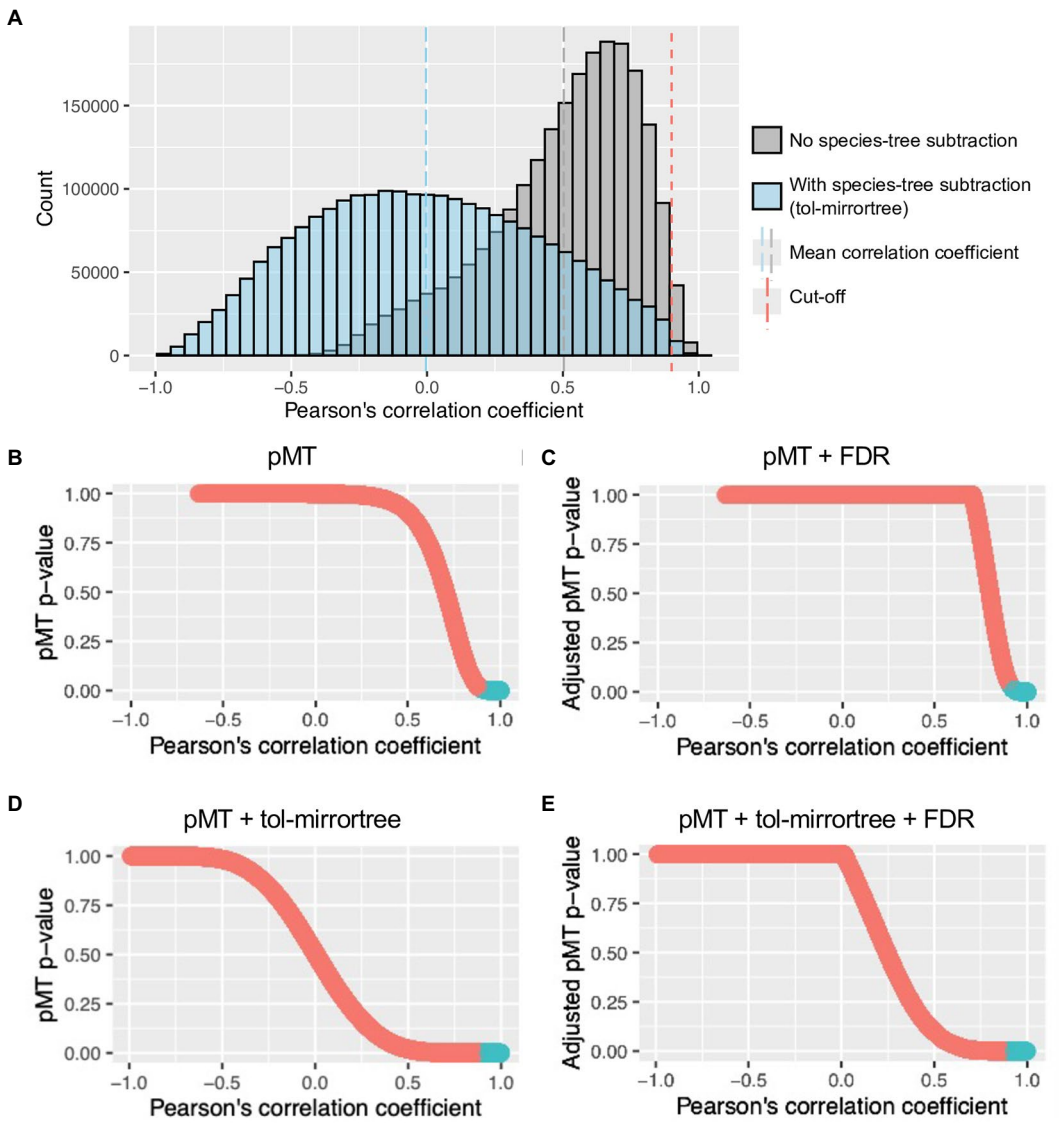
Distribution of Pearson’s correlation coefficient and pMT value of ps. **(A)** Distribution of Pearson’s correlation coefficient of *Wolbachia*–nematode gene tree comparisons with (blue) or without (gray) species tree subtraction (tol-mirrortree). Blue and gray dash lines are means of correlation coefficients with and without species tree subtraction, respectively. Red dash line indicates a cutoff value used for selecting top-scoring genes (Pearson’s correlation coefficient ≥ 0.9). **(B–E)** Relationship between Pearson’s correlation coefficient and (Benjamini–Hochberg adjusted) pMT *p*-value after different combination of corrections. Blue sections are *Wolbachia*–nematode correlation that pass both cutoff values (Pearson’s correlation coefficient ≥ 0.9 and adjusted pMT-*p*-value < 0.05).

To evaluate gene-level co-evolution between the nematode and *Wolbachia*, each of the 1-to-1 ortholog groups of the nematode was paired and correlated with those of the *Wolbachia*. The resulting Pearson’s correlation coefficient scores, without species tree subtraction, had a mean of 0.503 (range −0.632 to 1; Figure 2A), with a higher correlation coefficient indicating a stronger co-evolutionary signal. After applying a mirrortree-specific *p*-value (pMT) followed by the Benjamini–Hochberg method for multiple testing correction (adjusted pMT *p*-value <0.05) and Pearson’s correlation coefficient score cutoff value (*r* ≥ 0.90), 39,537 out of 2,428,734 pairs (1.6%) of nematode–*Wolbachia* genes were identified as top-scoring pairs (Figures 2B,C).

To reduce the influence of co-evolution at the species level on gene-level co-evolution, species trees for the nematode and the *Wolbachia* were created and their distance matrices were subtracted from each gene distance matrix of the nematodes or *Wolbachia*, respectively. Pearson’s correlation coefficient of the subtracted gene distance matrices ranged from −0.990 to 0.998, with a mean of −0.004 (Figure 2A). After applying pMT followed by the Benjamini–Hochberg method for multiple testing correction (adjusted *p*-value <0.05), 254,276 passed this adjusted *p*-value cutoff, but with a correlation coefficient score as low as 0.57 (Figures 2D,E). Based on the distribution of the correlation coefficient (Figure 2A), we further applied a correlation coefficient cutoff value of 0.90, resulting in a total of 8,285 pairs of nematode–*Wolbachia* genes being identified as top-scoring pairs (adjusted pMT *p*-value of <0.05, Pearson’s correlation coefficient > 0.90).

### 3.2. Top-scoring pairs revealed the co-evolution of gene expression regulation and multiple biosynthesis processes

The total top-scoring pairs from the tol-mirrortree + pMT method (8,285 pairs) were used for functional analyses to investigate the biological implications of these putatively co-evolving genes. Genes of *B. malayi* (Bm) or *Wolbachia* associated with *B. malayi* (wBm) were used as group representatives due to the high quality of the *B. malayi* genome compared to other filarial nematode species.

**Figure 3:**
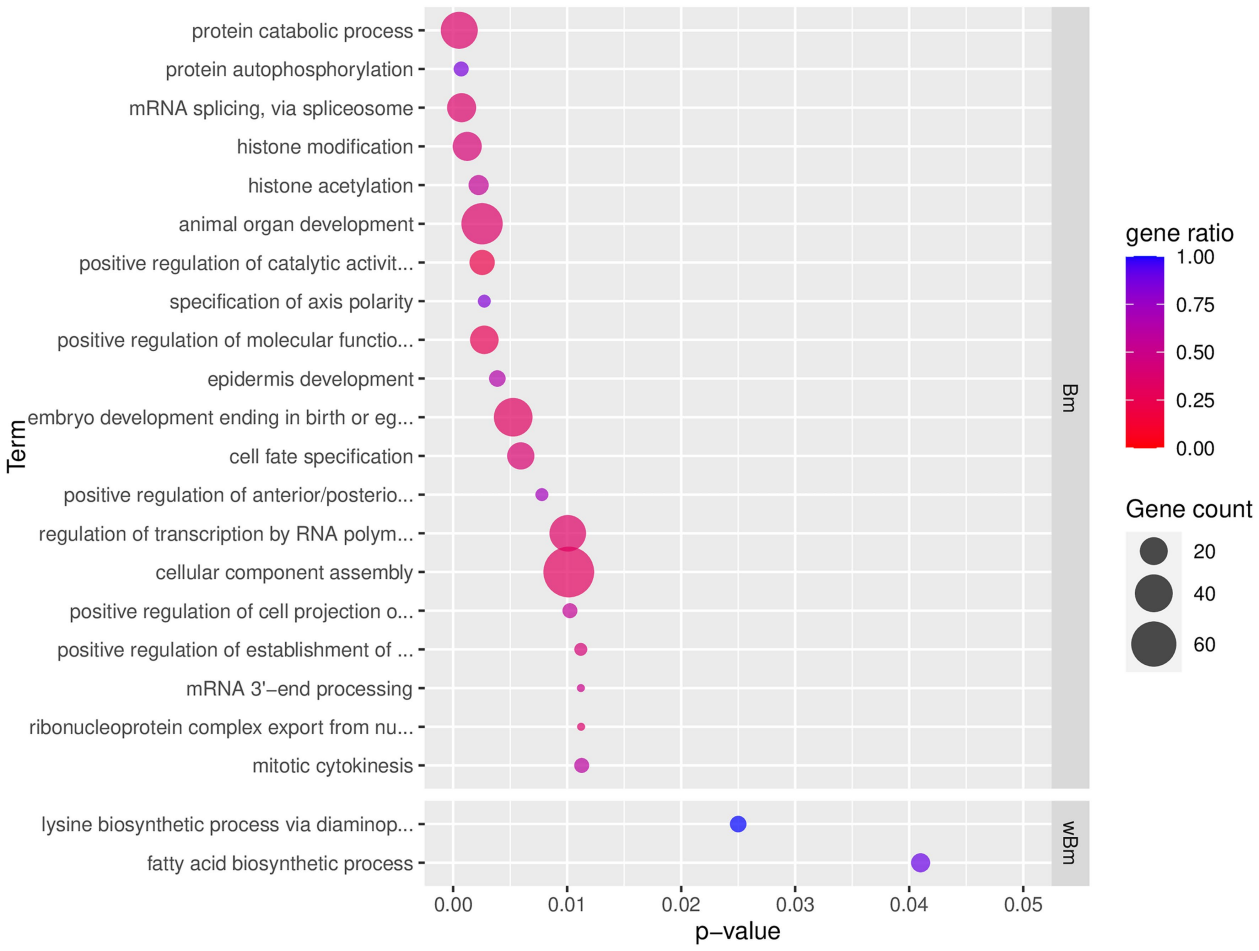
Top 20 enriched GO term of *B. malayi* and *Wolbachia* of *B. malayi* genes in top-scoring pairs. Only the top 20 enriched GO terms, ranked by *p*-value, of the biological processes type (BP) are shown. *p*-value refers to the *p*-value reported by topGO analysis. Gene count indicates the number of genes with that GO term in the input top-scoring gene list. Gene ratio is the number of genes with that GO term in the input list divided by a total number of genes in the genome annotated with that GO term. Full list of enriched GO terms of the BP type is presented in Supplementary Figure S1. The complete GO enrichment results with GO ID, full GO term description, and the top-scoring genes in each GO term are shown in Supplementary Table S1 (for *B. malayi*) and Supplementary Table S2 (for *Wolbachia* of *B. malayi*). *Wolbachia*’s lysine biosynthesis and fatty acid synthesis networks and their co-evolving genes in the nematode are further explored in Supplementary Figures S2, S3, respectively. Interactive networks can be found in Cytoscape session file (Supplementary File S1).

For *B. malayi*, 1,959 genes were present in the 8,285 top-scoring pairs (some genes were in multiple pairs; i.e., co-evolving with multiple *Wolbachia* genes). GO term enrichment of these genes revealed enrichment in biological processes related to phosphorylation, histone modification, developmental regulation, gene expression regulation, and mRNA processing (Figure 3; Supplementary Figure S1; Supplementary Table S1). For molecular functions, enriched GO terms were related to transferase activity, signaling *via* MAPK, calmodulin, GTP, serine/threonine phosphatase, translation, and ribosomal processes (Supplementary Table S1). The cellular component GO terms suggested events related to alternative splicing (*spliceosomal complex* and *U4/U6 x U5 tri-snRNP complex*), translation (*large ribosomal subunit*), and vesicle transport (*clathrin-coated vesicle*, *transport vesicle*, and *phagocytic vesicle*; Supplementary Table S1).

For *Wolbachia*, 386 genes were present in the top-scoring pairs. Only a small number of GO terms were identified as enriched in the *Wolbachia* genes in the top-scoring pairs. Nevertheless, the number of top-scoring genes that were found under these GO terms covered almost or all genes annotated with such GO terms (gene ratio ~ 1), suggesting a strong signal for functional enrichment (Figure 3; Supplementary Figure S1; Supplementary Table S2). These enriched GO terms were related to amino acid synthesis (*lysine biosynthetic process via diaminopimelate*; Supplementary Figure S2), *fatty acid biosynthesis* (Supplementary Figure S3), oxidation reduction (particularly with enzymes in fatty acid synthesis, TCA cycle, and co-factor biosynthesis), and peptidase activity (Supplementary Table S2).

### 3.3. Some co-evolving genes were an apparent hub of potential interactions

A number of gene orthologous groups were connected to multiple gene groups of the symbiotic partner. Focusing on the nematode, and using *B. malayi* as a group representative, the top 1% most highly connected nodes (20 nodes) were orthologous groups related to kinase and signaling activities including *stress-activated protein kinase JNK* (involved in various pathways related to stress responses and cell differentiation), *EGL-15* (contained tyrosine-protein kinase receptor domain), *Bm6186* (belonged to ribose-phosphate diphosphokinase family which is involved in catalyzing PRPP – an essential precursor for purine and pyrimidine biosynthesis), and *SOS-1* (involved in activation of RAS/MAPK pathway and regulation of growth, cell differentiation, and survival). Other groups among the top 1% can be related to the regulation of gene expression, translation, RNA pre-processing, and alternative splicing. These included *vab-3* (containing the homeobox domain), *NPAX-2* (predicted to affect transcription factor activity *via* DNA binding), *TBP-1* (TATA-box binding protein), *LIN-40* (predicted to have histone deacetylase binding activity), *MBD-2* (Methyl-CpG Binding Domain Protein), the 40S and 60S ribosomal proteins, *Integrator complex subunit 11* (involved in transcription of small RNA involved in spliceosome), *CWF19* (part of spliceosome), and *fcp-1* (involved in dephosphorylation of RNA polymerase II C-terminal domain) (Table 2; Supplementary Table S3; Supplementary Files S1, S2).

**Table 2:** Top 1% most highly connected nodes in the nematode and *Wolbachia.*

Gene ID		Gene information
*Wolbachia of B. malayi*	AAW70634	Gene: Wbm0042 (Aspartate-semialdehyde dehydrogenase)
	AAW70716	Gene: Wbm0125 (NADH:ubiquinone oxidoreductase chain D)
	AAW70865	Gene: Wbm0276 (ATPase involved in DNA replication initiation, DnaA)
	AAW71149	Gene: Wbm0561 (Dihydrolipoamide dehydrogenase E3 component)
*B. malayi*	WBGene00222081	Gene: Bma-jnk-1 (Stress-activated protein kinase JNK)
	WBGene00222891	Gene: Bm2630 (Cleft lip and palate associated transmembrane protein 1, putative)
	WBGene00223114	Gene: Bma-rps-2.1 (40S ribosomal protein S2)
	WBGene00223516	Gene: Bma-ints-11 (Integrator complex subunit 11)
	WBGene00223630	Gene: Bma-vab-3 (Variable ABnormal morphology, PAX6 ortholog)
	WBGene00223673	Gene: Bma-cwf-19 L1 (CwfJ C-terminus 1 containing protein)
	WBGene00224135	Gene: Bma-fcp-1 (RNA polymerase II subunit A C-terminal domain phosphatase)
	WBGene00224457	Gene: Bma-npax-2 (Paired domain-containing protein)
	WBGene00224470	Gene: Bm4209 (FAD_binding_2 domain-containing protein)
	WBGene00224578	Gene: Bma-unc-108 (Ras-related protein Rab-2A)
	WBGene00224796	Gene: Bma-egl-15 (fibroblast growth factor receptor EGL-15)
	WBGene00226117	Gene: Bma-tbp-1 (TATA-box-binding protein-1)
	WBGene00226303	Gene: Bma-lin-40 (metastasis associated 1 family member 2 ortholog)
	WBGene00226342	Gene: Bma-sos-1 (Son Of Sevenless Homolog 1)
	WBGene00226447	Gene: Bm6186 (ribose-phosphate diphosphokinase family member)
	WBGene00229500	Gene: Bma-rpl-7A.3 (60S ribosomal protein L7a)
	WBGene00230969	Gene: Bm10708/Bma-ztf-18.2 (Zinc Finger domain-containing)
	WBGene00231010	Gene: Bma-mbd-2 (methyl-CpG binding domain protein 2)
	WBGene00233173	Gene: Bm12912 (DET1 Partner Of COP1 E3 Ubiquitin Ligase)
	WBGene00234073	Gene: Bma-xpb-1 (General transcription and DNA repair factor IIH helicase subunit XPB)

Among *Wolbachia* orthologs, the top 1% most highly connected nodes (four nodes) included the *aspartate-semialdehyde dehydrogenase* (*ASD*) gene that encodes an essential enzyme in the biosynthesis of amino acids in bacteria (Harb and Abu, 1998) and an enzyme in the electron transport chain (*NADH:ubiquinone oxidoreductase chain D*; *nuoD*), the ATPase *dnaA* that activates the initiation of DNA replication, and *dihydrolipoamide dehydrogenase E3 component* (*DLD*) that forms a subunit of several enzyme complexes that are largely involved in energy production *via* breaking down of biomolecules (Table 2; Supplementary Table S3; Supplementary Files S1, S2).

**Figure 4:**
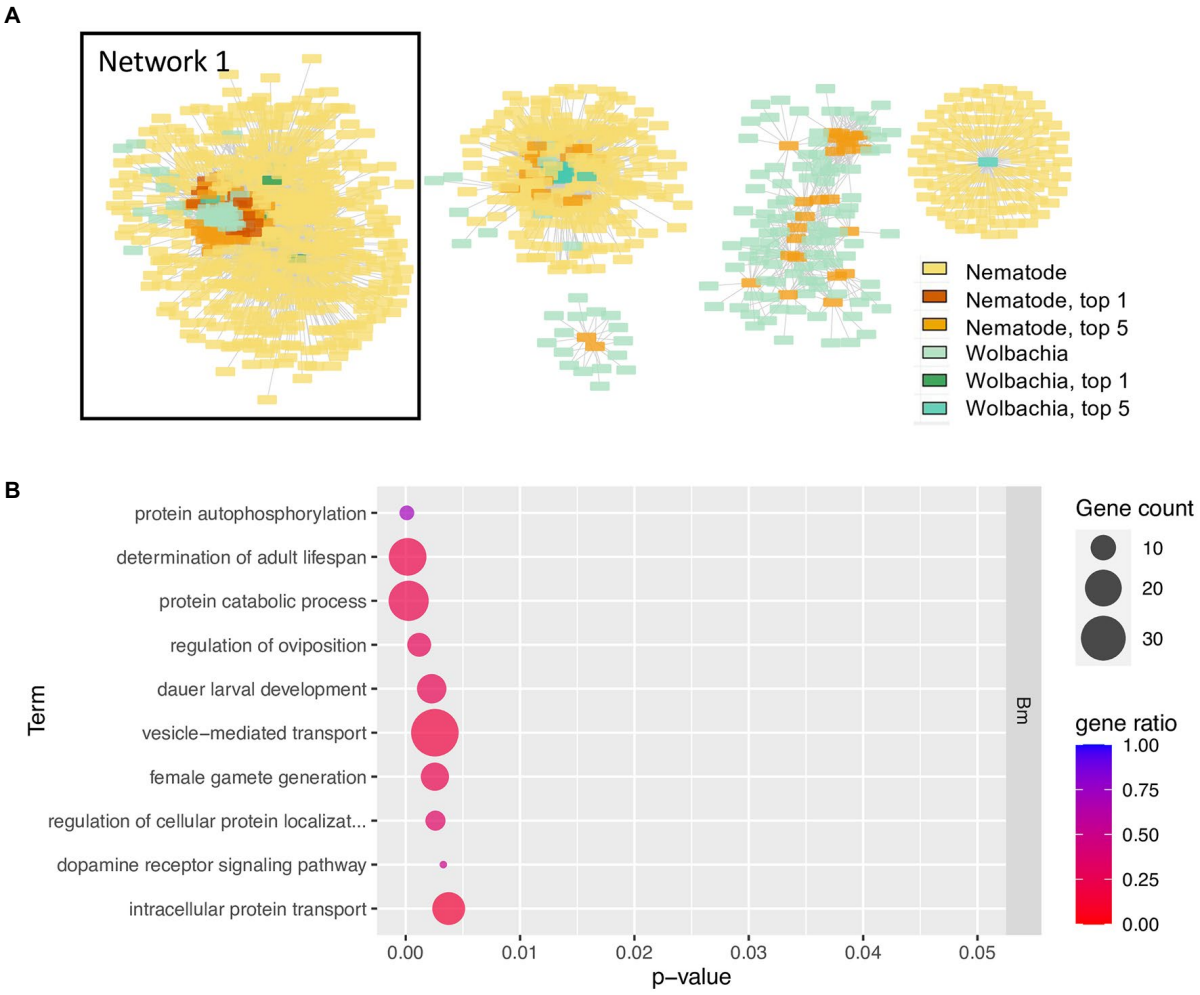
Network of top 5% most highly connected genes and enriched biological processes of Network 1 **(A)** The network contains the top 5% most highly connected genes of the *Wolbachia* and of the nematodes and their co-evolving genes in the symbiotic partner. Colors in yellow-red tone indicate genes in nematodes; blue–green tone indicates genes in *Wolbachia*, with deeper colors indicating the top 5% or top 1% most connected genes. List of all genes in Network 1 is provided in Supplementary Table S3. Interactive networks can be found in Cytoscape session file (Supplementary File S1). (B) Enriched GO terms of *B. malayi* genes in Network 1, a network that contains all top 1% most connected nodes. Only the top 10 enriched GO terms, ranked by *p*-value, of the biological processes type (BP) are shown. *p*-value refers to the *p*-value reported by topGO analysis. Gene count indicates the number of genes with that GO term in the input top-scoring gene list. Gene ratio is the number of genes with that GO term in the input list divided by a total number of genes in the genome annotated with that GO term. Full list of enriched GO terms, BP type, in Network 1, is presented in Supplementary Figure S4. The complete GO enrichment results for Network 1 with GO ID, full GO term description, and the top-scoring genes in each GO term are in Supplementary Table S4 (for *B. malayi*) and Supplementary Table S5 (for *Wolbachia* of *B. malayi*). Nematode’s genes in Network 1 with GO annotation *determination of adult lifespan* and their co-evolving genes in the *Wolbachia* are further explored in Supplementary Figure S5.

To investigate genes that may have co-evolved with multiple symbiotic partner’s genes, and hence could be hubs of the interactions, the top 5% most highly connected nodes of both nematode and *Wolbachia* and their first neighbors (orthologous groups of their symbiotic partner) were selected for producing a network (Figure 4A). Intriguingly, all of the top 1% most highly connected nodes were part of a single connected component, here referred to as Network 1 (Figure 4A). This Network 1 contained a total of 613 genes, with 66 genes being the top 5% most highly connected nodes (accounting for 65% of all the top 5% nodes; Supplementary Table S3). GO term enrichment of the genes in Network 1 revealed multiple processes and functions well-known for the *Wolbachia*–filarial nematode relationship, for example, *determination of adult lifespan*, *regulation of oviposition*, *dauer larval development*, *flavin adenine dinucleotide binding*, *NAD binding*, *mRNA splicing*, post-translational processes such as protein folding and localization, and various terms related to developmental control (Figure 4B). However, the enrichment results also point to functions not previously associated with *Wolbachia*–nematode interactions including *vesicle-mediated transport*, *histone acetylation*, *positive regulation of transcription by RNA polymerase II*, and *protein autophosphorylation*. For the *protein autophosphorylation* GO term, seven nematode genes across its genome were annotated with such GO term, with five of these found in Network 1, and the GO term appeared as the most significant enriched GO term (Figure 4B; Supplementary Figure S4; Supplementary Tables S4, S5).

**Figure 5:**
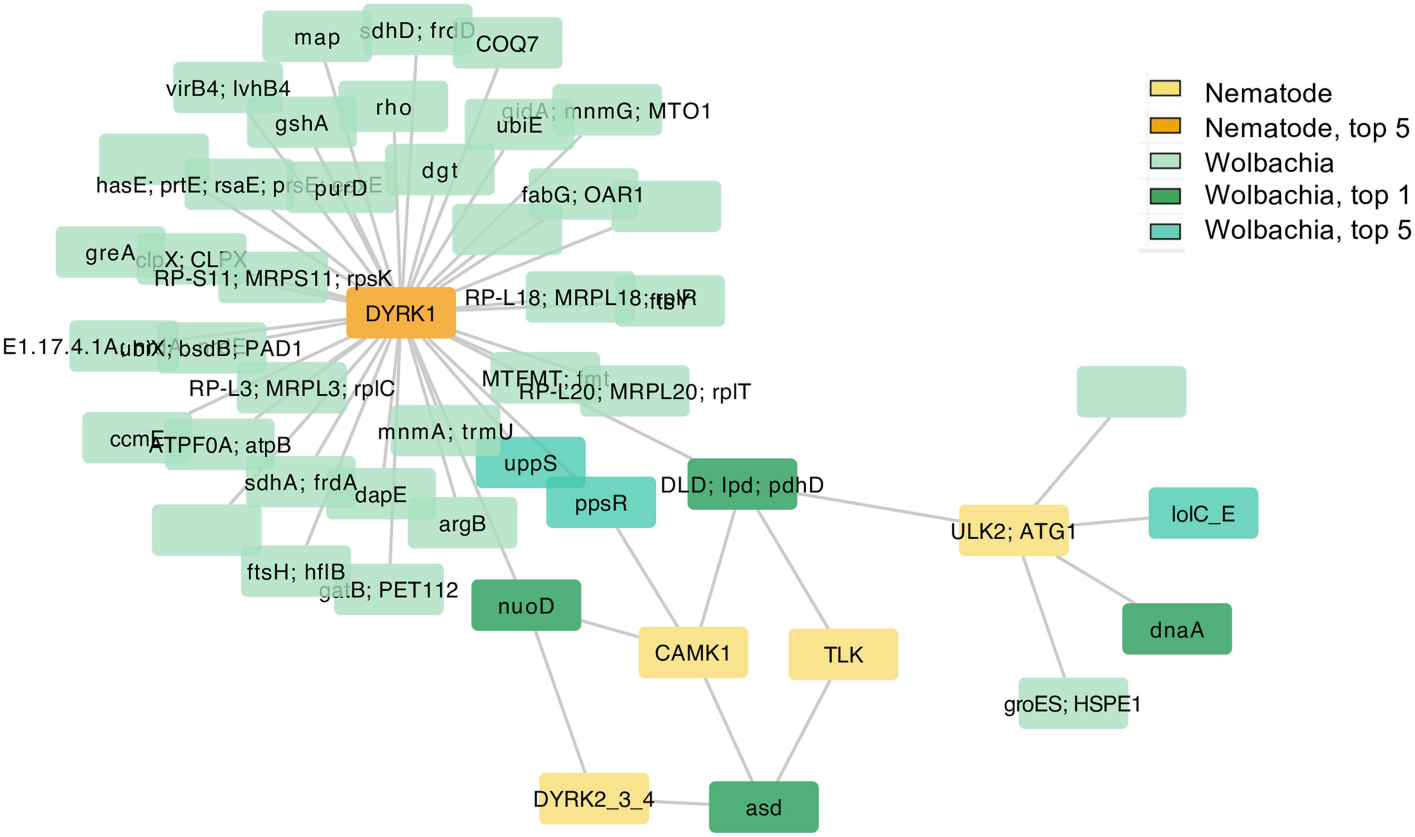
Genes in Network 1 that co-evolved with nematode genes annotated with GO term *protein autophosphorylation*. The nematode genes annotated with GO term *protein autophosphorylation* (five nodes in yellow and orange) and their first neighbors (*Wolbachia* genes) were selected from all genes that passed the cutoff value and viewed as a separated network here. Colors in yellow-red tone indicate genes in nematodes; blue–green tone indicates genes in *Wolbachia*, with deeper colors indicating the top 5% or top 1% most connected genes. Interactive networks can be found in Cytoscape session file (Supplementary File S1).

Upon investigation of the *protein autophosphorylation* GO terms, all of the five nematode genes were related to serine–threonine kinase and mitogen-activated protein kinase. These are *dual specificity tyrosine-phosphorylation-regulated kinase 1* (*DYRK1*), *tousled-like kinase* (*TLK*), *DYRK2/3/4*, *calcium/calmodulin-dependent protein kinase I* (*CAMK1*), and *serine/threonine-protein kinase ULK2* (*ULK2; ATG1*; Supplementary Table S4). Human orthologs of these serine/threonine kinases are known to be involved in the regulation of cell differentiation and proliferation, survival, development, autophagy, calcium signaling processes, DNA replication, transcription, DNA repair, and chromosome segregation (Safran et al., 2021). Even though four, out of five, of these nematode kinases were neither among the top 1% nor top 5% nodes, they showed a signal of co-evolution with all of the four *Wolbachia* top 1% nodes which were largely related to energy metabolism, amino acid biosynthesis, and DNA replication (Figure 5; Supplementary File S1).

In contrast, one of the kinases, *DYRK1,* was among the top 5% most highly connected nodes of the nematode genes (Figure 5; Supplementary File S1; Supplementary Table S3). This nematode DYRK1 appeared to co-evolve with some of the top 1% most connected genes in *Wolbachia* including *DLD* and *nuoD* genes. It was also connected to two top 5% most connected Wolbachia genes *undecaprenyl pyrophosphate synthase* (*UPPS*; involved in the production of precursor for bacterial cell wall biosynthesis) and *phosphoenolpyruvate synthase regulatory protein* (*ppsR*; another serine/threonine kinase, now in *Wolbachia*, that regulate the conversion of pyruvate to phosphoenolpyruvate (PEP) which can be used for gluconeogenesis or energy production).

In addition, other *Wolbachia* genes that appeared to co-evolve with the nematode *DYRK1* included an ATPase component *VirB4* of bacterial type 4 secretion system (T4SS), as well as a membrane fusion protein *hasE* which belongs to the type 1 secretion system (T1SS). These two secretion system components, *VirB4* (T4SS) and *HasE* (T1SS), also co-evolved with the nematode *p38* gene, also known as *mitogen-activated protein kinase 14* (*MAPK14*), which is involved in a huge variety of biological processes including cell differentiation, transcription regulation, development, and responses to stresses. *VirB4* (a T4SS component), *HasE* (a T1SS component), and *p38* were all in the network of genes connected to the second enriched GO term *determination of adult lifespan* (Figure 4B; Supplementary Table S4; Supplementary File S1). Among genes in this network, another secretion system component *HasD*/*AprD* gene was present which positions as an ATP-binding cassette in T1SS (Supplementary Figure S5; Supplementary File S1).

### 3.4. Candidate drug targets from the highly connected genes

In addition to revealing the potentially novel biology of *Wolbachia–*filarial nematode interaction, the highly connected nodes may also be useful as potential drug targets since disrupting their function may corrupt some of the key interactions between the nematode and its obligate symbiont, as well as affect the biological processes of the nematode or *Wolbachia* directly. To investigate the potential of nematode–*Wolbachia* co-evolving genes as novel drug targets, we searched for sequence similarity to known drug targets by screening the top 1% most highly connected nodes of *B. malayi* and *Wolbachia* of *B. malayi* using DrugBank Online^3^ (Wishart et al., 2006). Among the 24 genes comprising the top 1% of nodes used for the target search, eight sequences were similar to known drug targets with BLAST E-value of <0.00001, which was a default setting of DrugBank Online and a cutoff value used by other drug-target studies (e.g., Rajamanickam et al., 2020; Table 3; Supplementary Table S6).

**Table 3:** Target hits on DrugBank Online of the top 1% genes.

Top 1% nodes with matched targets on DrugBank	DrugBank top target	Top target *E*-value	Top target bit score	The number of drugs for all identified targets
AAW70716	NADH dehydrogenase [ubiquinone] iron–sulfur protein 2, mitochondrial (Humans)	0	561.222	2
AAW71149	Dihydrolipoyl dehydrogenase, mitochondrial (Humans)	5.28E−134	394.815	11
WBGene00222081	Mitogen-activated protein kinase 10 (Humans)	0	590.497	18
WBGene00223114	40S ribosomal protein S2 (Humans)	3.82E−134	379.407	1
WBGene00224470	Fumarate reductase flavoprotein subunit (*Shewanella frigidimarina*)	4.27E−73	242.276	2
WBGene00224578	Rho-related GTP-binding protein RhoB (Humans)	2.93E−21	85.8853	7
WBGene00224796	Fibroblast growth factor receptor 1 (Humans)	1.36E−122	401.364	96
WBGene00226447	Phosphoribosylpyrophosphate synthetase (*Plasmodium falciparum*)	1.82E−48	167.933	1

**Figure 6:**
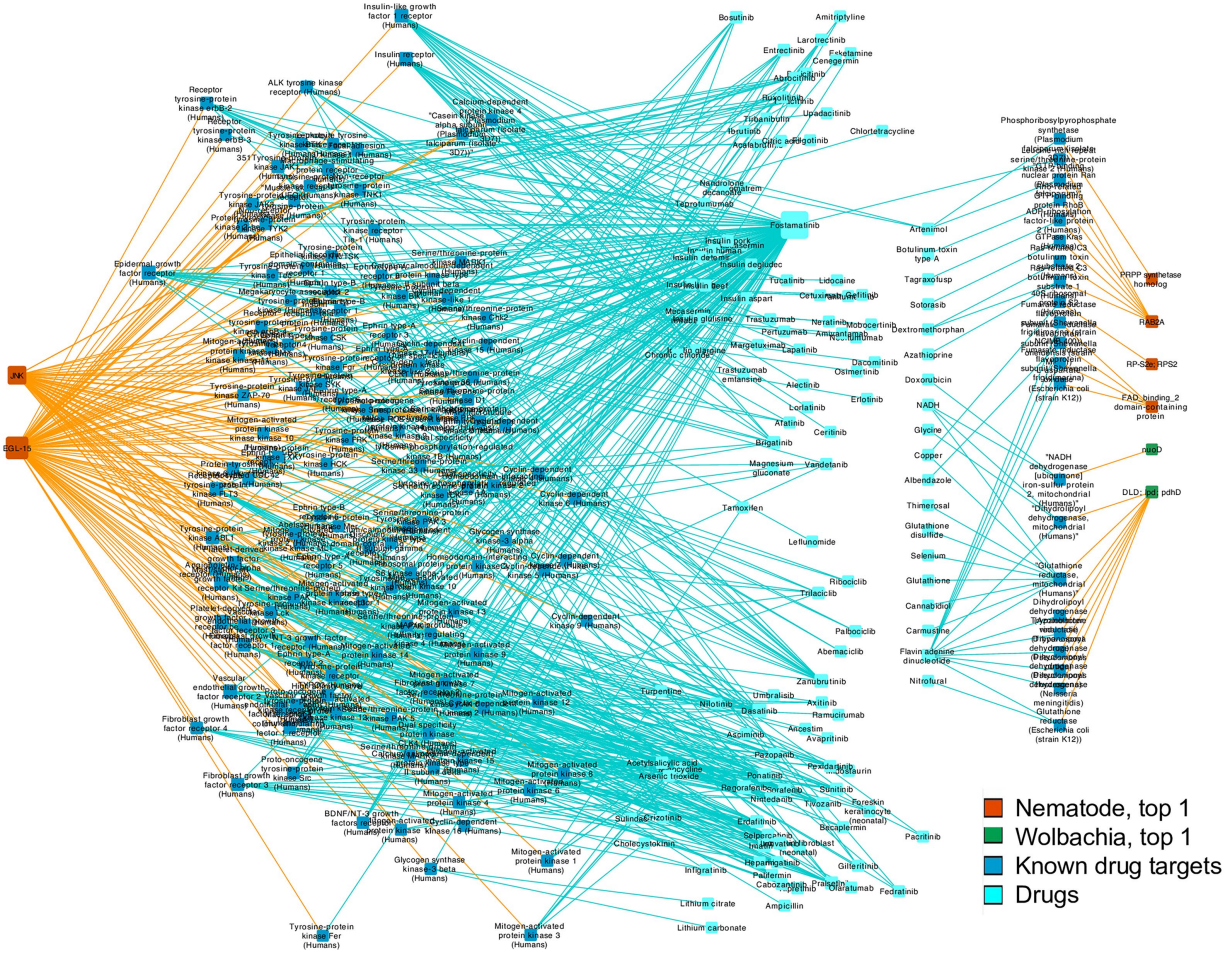
Drug-gene network Connections between genes from the top 1% most highly connected nodes of nematodes and *Wolbachia* genes, and their matched drug targets from DrugBank Online search, and related drugs acting on the drug targets based on DrugBank Online report. Gene nodes without a matched drug target are not shown. Node sizes indicate the degree of connections, i.e., how many edges are connected to a given node. Orange edges link genes in our study and known drug targets. Blue edges link known drug targets to their relevant drugs.

Full list of matched drug targets and related drugs is in Supplementary Table S6. Visualization of this data is in Figure 6 and Supplementary File S3.

Overall, the target sequence hits that pass the E-value and bit score cutoff value cover a range of common drug targets including enzymes, receptors, and binding proteins. Top hits to DrugBank targets include *NADH dehydrogenase*, *dihydrolipoyl dehydrogenase* (*DLD*), *mitogen-activated protein kinase 10*, *40S ribosomal protein S2*, *fumarate reductase flavoprotein subunit*, *Rho-related GTP-binding protein RhoB*, *fibroblast growth factor receptor 1*, and *phosphoribosylpyrophosphate (PRPP) synthetase* (Table 3; Figure 6; Supplementary Table S6; Supplementary File S3). The drugs identified included albendazole, a known broad-spectrum anthelmintic drug used against *Taenia solium* and *Echinococcus granulosus*, which was coupled with antibiotics for killing filarial nematodes (Turner et al., 2017; Laman et al., 2022). Moreover, two of the nematode kinases were similar to multiple kinases in the DrugBank Online database. Kinases are widely targeted enzymes in many diseases including cancer, immune-related diseases, and infections. As a result, multiple drugs that are small molecule kinase inhibitors were identified in our search, such as imatinib, nilotinib, dasatinib, and fostamatinib (Table 3; Figure 6; Supplementary Table S6; Supplementary File S3).

## 4. Discussion

In this study, we performed a genome-wide co-evolutionary analysis between gene families of the filarial nematode and their obligate *Wolbachia* symbiont using publicly available genome data to seek protein–protein interactions that could be keys for their symbiotic relationship. The results showed that co-evolving genes were consistent with the known metabolic processes between the nematode–*Wolbachia* interactions and suggested gene-level details as well as potentially novel interactions. The co-evolving genes were involved in a range of metabolic and signaling processes including amino acid, lipid, carbohydrate, and nucleotide metabolism, regulation of transcription through transcription factor binding and epigenetic modification, pre-mRNA splicing, post-translational modification, signal transduction, and bacterial transport system. Some genes may be co-evolving with multiple other genes, suggesting potentially prominent roles in the interactions. Moreover, highly connected, co-evolving genes share sequence similarity and protein domains highly similar to known targets of approved drugs. It is worth noting that some of these drugs may also affect human drug target homologs and, therefore, their potential side effects should be taken into account. This information may help prioritize candidate targets that can be further tested for drug re-purposing and can lead to better treatment for at-risk populations of filariasis worldwide.

Due to their essential regulatory roles in various biological processes, protein kinases have been proposed as drug targets against parasitic infections, from protozoa to helminths (Beckmann and Grevelding, 2010; O’Connell et al., 2015; Kesely et al., 2016; Wu et al., 2021). In our data, two kinases were among the top 1% of most highly connected genes and showed high similarity to known drug targets. In addition, multiple serine/threonine kinases were part of the network that contained all of the top 1% most connected genes. Three of the kinase inhibitors identified on DrugBank online, i.e., imatinib, nilotinib, and dasatinib, have been tested against microfilaria, L3, and adult *B. malayi* and resulted in reduced survival rates in all stages (O’Connell et al., 2015). In addition, *MAPK*, *FGFR*, *SOS*, and *JNK*, all in the top 1% nodes of the nematode and each of them connected to overlapping sets of *Wolbachia* genes, are part of MAPK signaling pathways, which are generally known to control cell proliferation (Avruch et al., 2001) and have roles in pro-inflammatory pathogenesis in patients with filariasis (Babu et al., 2011).

*Wolbachia* and filarial nematodes rely on metabolic substrate provisioning from one another (Taylor et al., 2013). For example, filarial nematode spp. is known to possess the pathway for producing PRPP, but the steps from PRPP to the production of purine precursor (IMP) turn out to be incomplete (International Helminth Genomes Consortium, 2019). *Wolbachia* is thought to supply nucleotides to its nematode host and to take pyruvate and amino acids from the nematode to use it as an energy source and for the production of other biomolecules (Foster et al., 2005; Taylor et al., 2013; Voronin et al., 2019). Consistent with this knowledge, our data revealed, among the top 1% most connected nodes, a putative *Wolbachia DLD* gene, which encodes a component participating in breaking down amino acids (BCKD enzyme), and conversion between pyruvate and acetyl-CoA (pyruvate dehydrogenase), both can lead to the release of energy. Our dataset suggested that *Wolbachia* DLD may be co-evolving with nematode genes involved in RNA, protein, and nucleotide synthesis, all of which require energy input. In addition, *Wolbachia DLD* appeared to co-evolve with nematode’s putative *PRPP synthetase*, an essential enzyme for the production of PRPP, a key precursor for purine and pyrimidine biosynthesis. The potential connections between intermediate enzymes in amino acid and pyruvate metabolism in *Wolbachia* and biosynthesis in filaria suggested that their metabolic interactions may be more complex than previously thought.

Another gene among the top 1% in *Wolbachia* was annotated as *NADH dehydrogenase Fe-S* which forms part of Complex I in the electron transport chain. From our data, it may co-evolve with a nematode gene *fumarate reductase flavoprotein subunit*, which catalyzes the conversion of succinate to fumarate and forms part of Complex II in the electron transport chain. Previous data suggested that *Wolbachia* may aid its host in generating ATP *via* a mitochondrial-like function in somatic tissue based on high expression of ATPase and components of the electron transport chain (Darby et al., 2012). Our data further provide specific players that may be involved, suggesting a possibility that Complex I and Complex II may be interacting across species. Furthermore, Complex I and Complex II systems in filaria were previously proposed as potential targets, particularly the fumarate reductase protein, which was a known target for albendazole/benzimidazole (Barrowman et al., 1984; Gupta and Srivastava, 2005; Ahmad and Srivastava, 2007).

The involvement of fatty acid synthesis in the filarial nematode–*Wolbachia* relationship is not well understood. However, *Wolbachia* can affect lipid profiles in arthropod host and can modulate viral infection (Caragata et al., 2013; Molloy et al., 2016). Nematode and bacteria are capable of glyoxylation shunting which shortcuts the TCA cycle and utilizes fatty acid as a source of acetyl-CoA for gluconeogenesis and energy metabolism (International Helminth Genomes Consortium, 2019; Curran et al., 2020). Fatty acid would be required for growth and reproduction, and it is also an essential precursor for the production of steroid hormones including reproductive hormones. Our data showed that multiple *Wolbachia* genes in the fatty acid biosynthesis pathway may co-evolve with highly connected genes in the filarial nematode. The roles of fatty acid synthesis in filarial nematode–*Wolbachia* interaction may warrant further investigation.

Finally, much of the literature on filarial nematodes and *Wolbachia* focuses on the involvement of T4SS, which facilitate the transport of molecules from bacteria to the inside of host cells. In particular, T4SS is a major exchanger of effector proteins and small metabolites such as nucleotides and their precursors, and it is involved in the control of gene expression and germline development in filarial nematodes (Rancès et al., 2008; Slatko et al., 2010; Darby et al., 2012; Li and Carlow, 2012; Carpinone et al., 2018; Lindsey, 2020; Chevignon et al., 2021). Our data identified an ATPase component of the T4SS and also two components of the T1SS, another key bacterial secretion system that translocates proteins across the outer membrane into the extracellular space. T1SS is rarely investigated in the *Wolbachia*–filarial nematode interactions. However, the system is widespread in gram-negative bacteria and is involved in the secretion of peptidase, lipase, and toxin, as well as drug efflux (Thomas et al., 2014; Morgan et al., 2017; Kanonenberg et al., 2018). One of the genes identified in our analysis, the *HasD*/*AprD* gene, is important in *Pseudomonas fragi*, another Pseudomonadota gram-negative bacteria in the same phylum as *Wolbachia*, for its secretion of protease (Wang et al., 2021). A recent study showed that *Wolbachia* is important for microfilaria exsheathment, a process that requires proteolysis (Quek et al., 2022). Although less well-studied, T1SS is present in *Wolbachia* of both insects and filarial nematodes (Lindsey, 2020). The apparent co-evolution of T1SS components with various kinase enzymes, and with genes regulating development, stress response, and survival, suggests that the less studied T1SS could be an interesting novel avenue for *Wolbachia–*nematode communication and the signaling pathways that the communication may trigger.

Our approach has revealed gene sets that are relevant to the known biology between the filarial nematode and its *Wolbachia*. It has also allowed us to suggest interesting novel targets for further study. However, its power is limited to those proteins with one-to-one orthologs in each species which show a clear signal of co-evolution given our particular methodology. This is probably not the case for the majority of proteins that interact between the host and symbiont. Moreover, genes that had paralogs or were absent in at least one species or were missing due to their stage of the reference genome were excluded from the analyses. This may explain why our result, although pointing to relevant biological pathways and processes, was able to identify only a small number of genes in some pathways.

In addition to providing candidate drug targets and a list of conceivably repurposable drugs, this study paved a number of avenues, whereby the interactions between *Wolbachia* and filarial nematodes could be further investigated. Future validation of the interactions can utilize spatial information of the predicted interacting pairs and tracking of metabolic intermediates. The list of potential drugs and their targets may guide future investigations aimed at enhancing drug specificity toward filarial or *Wolbachia* proteins. Importantly, our analysis was based on genes shared across multiple filarial nematode species; hence, the implication provided is expected to be applicable to a wide range of filariasis diseases. Improvements in the availability of genome sequences for further filarial nematode species and their *Wolbachia* have the potential to improve the power of our approach, and it could be easily applied to other host-symbiont or host-pathogen systems.

## Data availability statement

Publicly available datasets were analyzed in this study. This data can be found here: Table 1. The codes used for data analysis were deposited at: https://github.com/akoiwang/filaria-wolbachia-coevolution.

## Author contributions

AW: conceptualization, data curation, funding acquisition, formal analysis, investigation, methodology, visualization, project administration, writing–original draft preparation, and writing–review and editing. SK: data curation, formal analysis, investigation, and visualization. JP: formal analysis, investigation, methodology, visualization, and writing–review and editing. NN: formal analysis, investigation, resources, and writing–review and editing. PK: formal analysis, investigation, and writing–review and editing. WL: formal analysis, investigation, resources, and writing–review and editing. WM: funding acquisition, resources, and supervision. AR: conceptualization, resources, software, validation, and supervision. All authors contributed to the article and approved the submitted version.

## Funding

This study was supported by the Thailand Research Fund (TRF) and the Office of the Higher Education Commission (OHEC) (MRG6280068).

## Conflict of interest

The authors declare that the research was conducted in the absence of any commercial or financial relationships that could be construed as a potential conflict of interest.

## Publisher’s note

All claims expressed in this article are solely those of the authors and do not necessarily represent those of their affiliated organizations, or those of the publisher, the editors and the reviewers. Any product that may be evaluated in this article, or claim that may be made by its manufacturer, is not guaranteed or endorsed by the publisher.

## Footnotes

^1^ https://github.com/adamjamesreid/hepatocystis-genome

^2^ https://go.drugbank.com/

^3^ https://go.drugbank.com/
