# Supplementary material for: Toward novel treatment against filariasis: Insight into genome-wide co-evolutionary analysis of filarial nematodes and Wolbachia
Source: Front Microbiol. 2023 Mar 22;14:1052352. doi: 10.3389/fmicb.2023.1052352 (PMC10073474; doi:10.3389/fmicb.2023.1052352)
Supplement: Supplementary file 1 [file Presentation_1.zip › File S2.pdf]

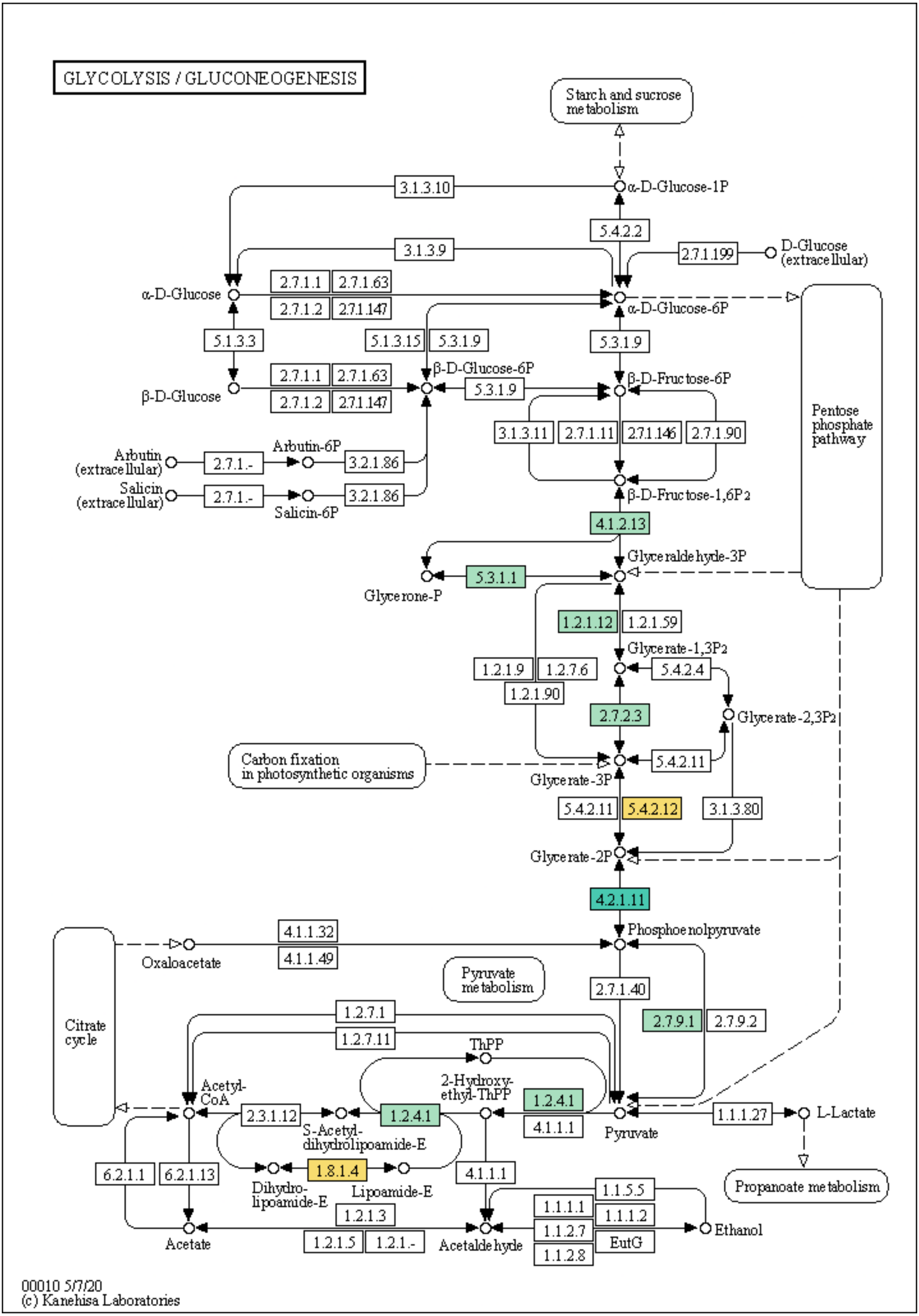
GLYCOLYSIS / GLUCONEOGENESIS
Starch and sucrose metabolism
α-D-Glucose-1P
3.1.3.10
5.4.2.2
3.1.3.9
2.7.1.199
D-Glucose (extracellular)
α-D-Glucose
2.7.1.1
2.7.1.63
2.7.1.2
2.7.1.147
α-D-Glucose-6P
5.1.3.3
5.1.3.15
5.3.1.9
5.3.1.9
β-D-Glucose-6P
β-D-Glucose
2.7.1.1
2.7.1.63
2.7.1.2
2.7.1.147
5.3.1.9
β-D-Fructose-6P
Pentose phosphate pathway
3.1.3.11
2.7.1.11
2.7.1.146
2.7.1.90
Arbutin (extracellular)
2.7.1.-
Arbutin-6P
3.2.1.86
Salicin (extracellular)
2.7.1.-
Salicin-6P
3.2.1.86
β-D-Fructose-1,6P2
4.1.2.13
Glyceraldehyde-3P
5.3.1.1
Glycerone-P
1.2.1.12
1.2.1.59
Glycerate-1,3P2
1.2.1.9
1.2.7.6
1.2.1.90
5.4.2.4
Glycerate-2,3P2
2.7.2.3
Carbon fixation in photosynthetic organisms
5.4.2.11
Glycerate-3P
5.4.2.11
5.4.2.12
3.1.3.80
Glycerate-2P
4.2.1.11
Phosphoenolpyruvate
4.1.1.32
4.1.1.49
Oxaloacetate
Citrate cycle
Pyruvate metabolism
2.7.1.40
2.7.9.1
2.7.9.2
1.2.7.1
1.2.7.11
ThPP
2-Hydroxy-ethyl-ThPP
1.2.4.1
1.2.4.1
4.1.1.1
Acetyl-CoA
2.3.1.12
S-Acetyl-dihydrolipoamide-E
Pyruvate
1.1.1.27
L-Lactate
Propanoate metabolism
6.2.1.1
6.2.1.13
1.8.1.4
Dihydro-lipoamide-E
Lipoamide-E
4.1.1.1
1.1.5.5
1.1.1.1
1.1.1.2
1.2.1.3
1.1.2.7
Ethanol
Acetate
1.2.1.5
1.2.1.-
Acetaldehyde
EutG
1.1.2.8
00010 5/7/20
(c) Kanehisa Laboratories

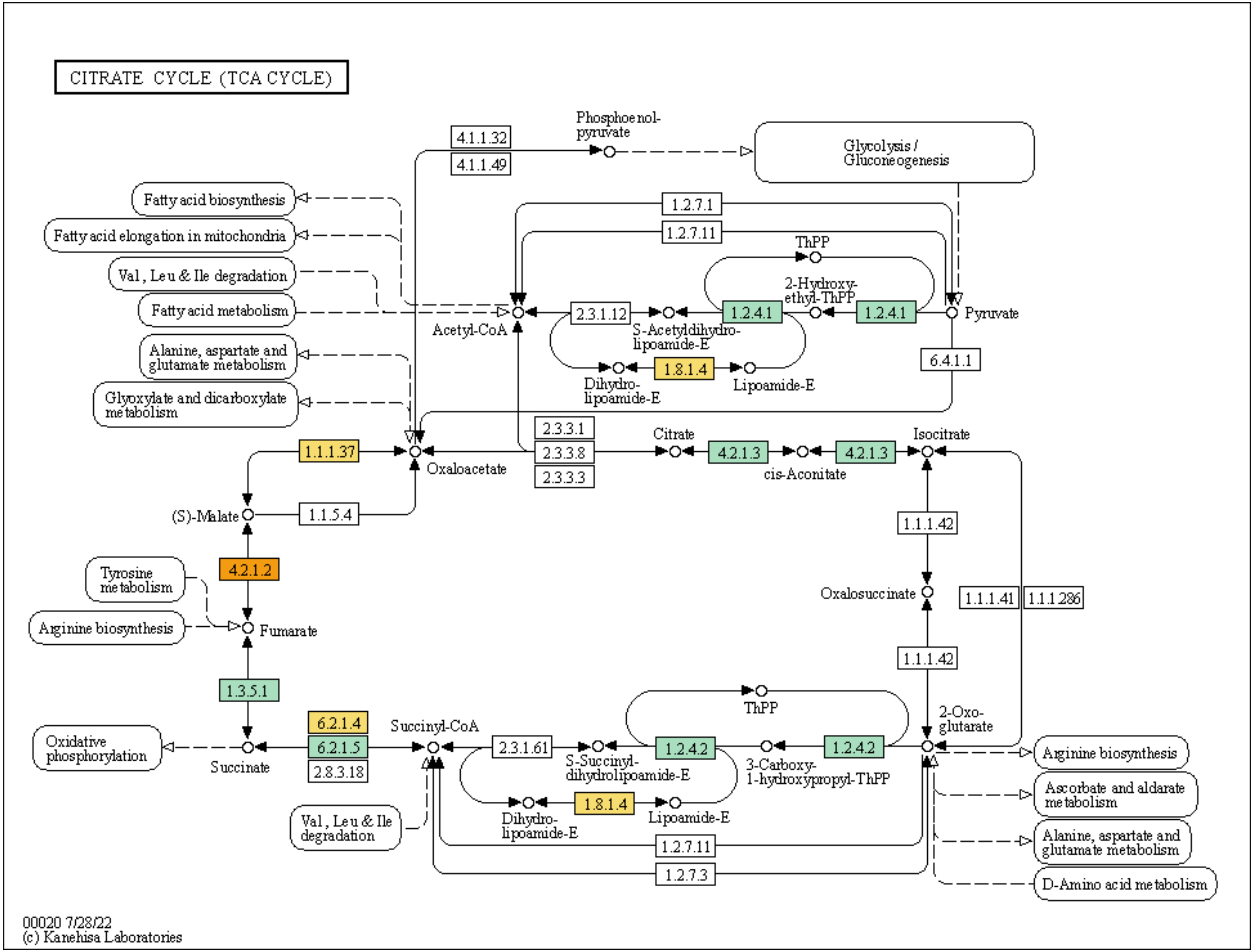
CITRATE CYCLE (TCA CYCLE)
4.1.1.32
4.1.1.49
Phosphoenol-pyruvate
Glycolysis / Gluconeogenesis
Fatty acid biosynthesis
Fatty acid elongation in mitochondria
Val, Leu & Ile degradation
Fatty acid metabolism
Alanine, aspartate and glutamate metabolism
Glyoxylate and dicarboxylate metabolism
1.2.7.1
1.2.7.11
ThPP
2-Hydroxy-ethyl-ThPP
Acetyl-CoA
2.3.1.12
S-Acetyldihydro-lipoamide-E
1.2.4.1
1.2.4.1
Pyruvate
Dihydro-lipoamide-E
1.8.1.4
Lipoamide-E
6.4.1.1
2.3.3.1
2.3.3.8
2.3.3.3
Citrate
4.2.1.3
cis-Aconitate
4.2.1.3
Isocitrate
1.1.1.37
Oxaloacetate
(S)-Malate
1.1.5.4
1.1.1.42
4.2.1.2
Oxalosuccinate
1.1.1.41
1.1.1.286
Tyrosine metabolism
Arginine biosynthesis
Fumarate
1.1.1.42
1.3.5.1
ThPP
2-Oxo-glutarate
6.2.1.4
6.2.1.5
2.8.3.18
Succinyl-CoA
2.3.1.61
S-Succinyl-dihydrolipoamide-E
1.2.4.2
3-Carboxy-1-hydroxypropyl-ThPP
1.2.4.2
Oxidative phosphorylation
Succinate
Dihydro-lipoamide-E
1.8.1.4
Lipoamide-E
Val, Leu & Ile degradation
1.2.7.11
1.2.7.3
Arginine biosynthesis
Ascorbate and aldarate metabolism
Alanine, aspartate and glutamate metabolism
D-Amino acid metabolism
00020 7/28/22
(c) Kanehisa Laboratories

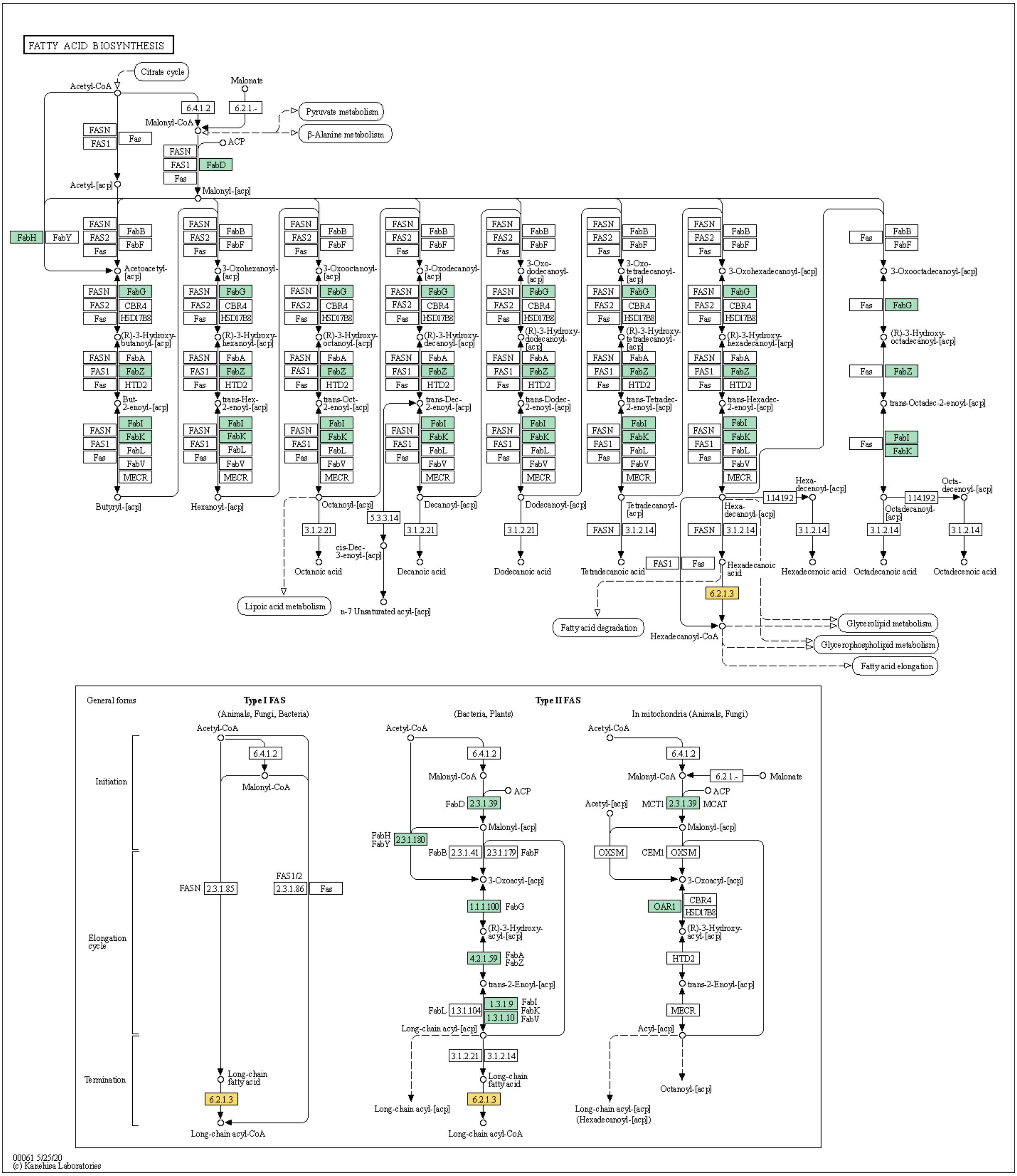
FATTY ACID BIOSYNTHESIS
Citrate cycle
Acetyl-CoA
Malonate
6.4.1.2
6.2.1.-
Pyruvate metabolism
Malonyl-CoA
β-Alanine metabolism
ACP
FASN
FAS1
Fas
FAS2
FabD
Acetyl-[acp]
Malonyl-[acp]
FabH
FabY
FabB
FabF
FabG
CBR4
HSD17B8
FabA
FabZ
HTD2
FabI
FabK
FabL
FabV
MECR
Acetoacetyl-[acp]
(R)-3-Hydroxy-butanoyl-[acp]
But-2-enoyl-[acp]
Butyryl-[acp]
3-Oxohexanoyl-[acp]
(R)-3-Hydroxy-hexanoyl-[acp]
trans-Hex-2-enoyl-[acp]
Hexanoyl-[acp]
3-Oxooctanoyl-[acp]
(R)-3-Hydroxy-octanoyl-[acp]
trans-Oct-2-enoyl-[acp]
Octanoyl-[acp]
3-Oxodecanoyl-[acp]
(R)-3-Hydroxy-decanoyl-[acp]
trans-Dec-2-enoyl-[acp]
Decanoyl-[acp]
3-Oxo-dodecanoyl-[acp]
(R)-3-Hydroxy-dodecanoyl-[acp]
trans-Dodec-2-enoyl-[acp]
Dodecanoyl-[acp]
3-Oxo-tetradecanoyl-[acp]
(R)-3-Hydroxy-tetradecanoyl-[acp]
trans-Tetradec-2-enoyl-[acp]
Tetradecanoyl-[acp]
3-Oxohexadecanoyl-[acp]
(R)-3-Hydroxy-hexadecanoyl-[acp]
trans-Hexadec-2-enoyl-[acp]
Hexadecanoyl-[acp]
3-Oxooctadecanoyl-[acp]
(R)-3-Hydroxy-octadecanoyl-[acp]
trans-Octadec-2-enoyl-[acp]
Octadecanoyl-[acp]
Hexa-decenoyl-[acp]
Octa-decenoyl-[acp]
1.14.19.2
3.1.2.21
3.1.2.14
5.3.3.14
cis-Dec-3-enoyl-[acp]
n-7 Unsaturated acyl-[acp]
Octanoic acid
Decanoic acid
Dodecanoic acid
Tetradecanoic acid
Hexadecanoic acid
Hexadecenoic acid
Octadecanoic acid
Octadecenoic acid
Lipoic acid metabolism
6.2.1.3
Fatty acid degradation
Hexadecanoyl-CoA
Glycerolipid metabolism
Glycerophospholipid metabolism
Fatty acid elongation
General forms
Type I FAS
(Animals, Fungi, Bacteria)
Type II FAS
(Bacteria, Plants)
In mitochondria (Animals, Fungi)
Initiation
Elongation cycle
Termination
FASN 2.3.1.85
FAS1/2 2.3.1.86 Fas
Long-chain fatty acid
Long-chain acyl-CoA
FabD 2.3.1.39
FabH FabY 2.3.1.180
FabB 2.3.1.41 2.3.1.179 FabF
3-Oxoacyl-[acp]
1.1.1.100 FabG
(R)-3-Hydroxy-acyl-[acp]
4.2.1.59 FabA FabZ
trans-2-Enoyl-[acp]
FabL 1.3.1.104 1.3.1.9 1.3.1.10 FabI FabK FabV
Long-chain acyl-[acp]
MCT1 2.3.1.39 MCAT
OXSM
CEM1 OXSM
OAR1
Acyl-[acp]
Octanoyl-[acp]
(Hexadecanoyl-[acp])
00061 5/25/20
(c) Kanehisa Laboratories

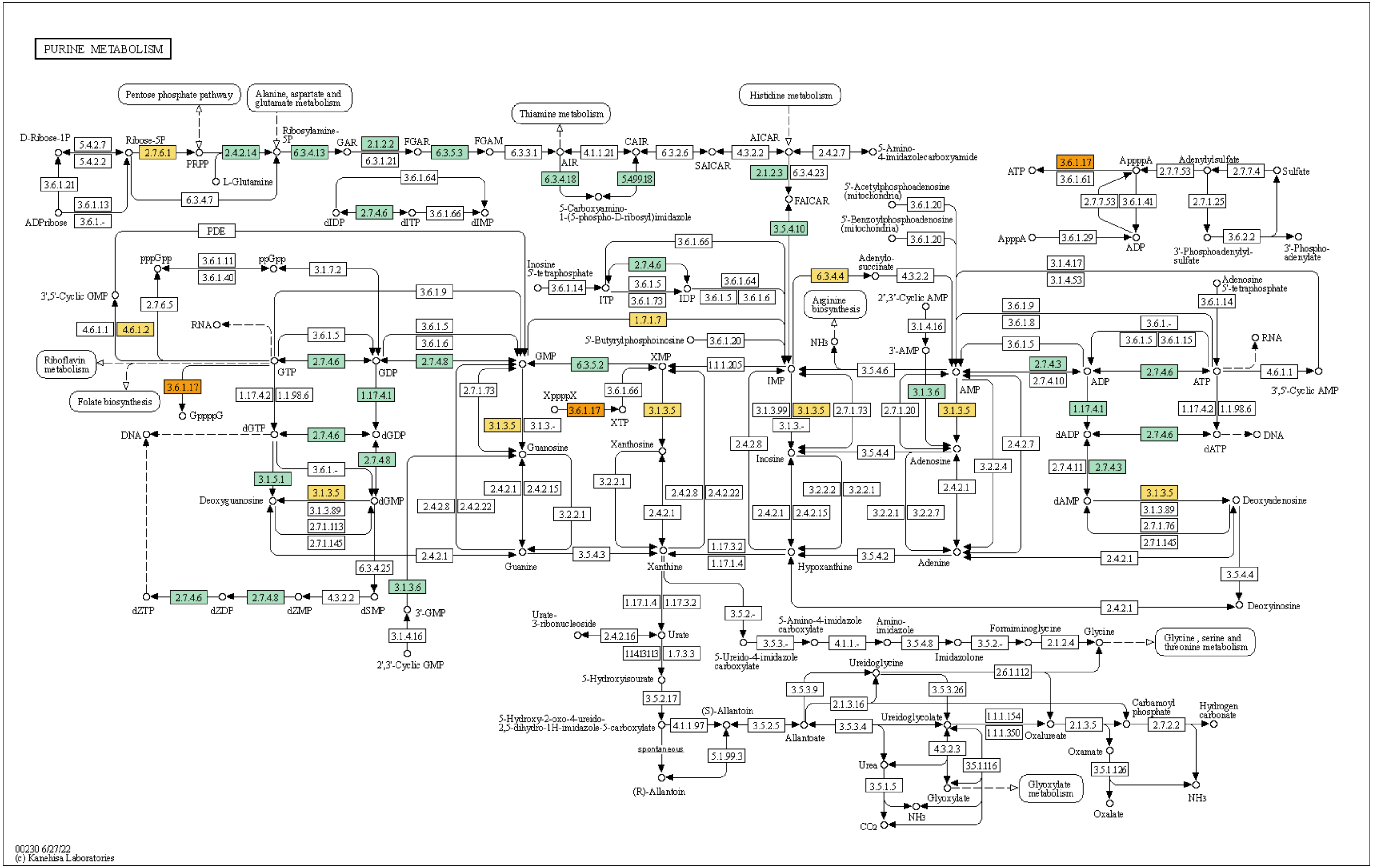

PURINE METABOLISM
Pentose phosphate pathway
Alanine, aspartate and glutamate metabolism
Thiamine metabolism
Histidine metabolism
D-Ribose-1P
5.4.2.7
5.4.2.2
Ribose-5P
2.7.6.1
PRPP
2.4.2.14
Ribosylamine-5P
6.3.4.13
GAR
2.1.2.2
6.3.1.21
FGAR
6.3.5.3
FGAM
6.3.3.1
AIR
4.1.1.21
CAIR
6.3.2.6
SAICAR
4.3.2.2
AICAR
2.4.2.7
5-Amino-4-imidazolecarboxyamide
L-Glutamine
6.3.4.7
3.6.1.21
3.6.1.13
3.6.1.-
ADPribose
6.3.4.18
5.4.99.18
5-Carboxyamino-1-(5-phospho-D-ribosyl)imidazole
2.1.2.3
6.3.4.23
FAICAR
3.5.4.10
3.6.1.64
dIDP
2.7.4.6
dITP
3.6.1.66
dIMP
PDE
3',5'-Cyclic GMP
pppGpp
3.6.1.11
3.6.1.40
ppGpp
3.1.7.2
2.7.6.5
4.6.1.1
4.6.1.2
RNA
Riboflavin metabolism
Folate biosynthesis
3.6.1.17
GppppG
GTP
3.6.1.5
3.6.1.9
3.6.1.6
GDP
2.7.4.8
GMP
6.3.5.2
XMP
1.1.1.205
1.17.4.2
1.1.98.6
1.17.4.1
DNA
dGTP
dGDP
3.1.5.1
3.6.1.-
Deoxyguanosine
3.1.3.5
3.1.3.89
2.7.1.113
2.7.1.145
dGMP
6.3.4.25
4.3.2.2
dSMP
dZMP
dZDP
dZTP
3.1.3.6
3'-GMP
3.1.4.16
2',3'-Cyclic GMP
2.7.1.73
3.1.3.-
Guanosine
2.4.2.1
2.4.2.15
2.4.2.8
2.4.2.22
3.2.2.1
Guanine
3.5.4.3
XppppX
3.6.1.17
XTP
Xanthosine
3.2.2.1
2.4.2.1
Xanthine
1.17.3.2
1.17.1.4
Inosine 5'-triphosphate
3.6.1.14
ITP
3.6.1.5
3.6.1.73
IDP
3.6.1.64
3.6.1.6
1.7.1.7
5'-Butyrylphosphoinosine
3.6.1.20
IMP
3.1.3.99
2.7.1.73
3.1.3.-
2.4.2.8
Inosine
3.5.4.4
3.2.2.2
2.4.2.1
2.4.2.15
Hypoxanthine
3.5.4.2
6.3.4.4
Adenylo-succinate
4.3.2.2
Arginine biosynthesis
NH3
5'-Acetylphosphoadenosine (mitochondria)
5'-Benzoylphosphoadenosine (mitochondria)
2',3'-Cyclic AMP
3.1.4.16
3'-AMP
3.5.4.6
AMP
2.7.1.20
Adenosine
3.2.2.4
2.4.2.7
3.2.2.1
3.2.2.7
Adenine
ATP
3.6.1.17
3.6.1.61
ApppA
Adenylylsulfate
2.7.7.53
2.7.7.4
Sulfate
3.6.1.41
2.7.1.25
ApppA
3.6.1.29
ADP
3'-Phosphoadenylyl-sulfate
3.6.2.2
3'-Phospho-adenylate
3.1.4.17
3.1.4.53
Adenosine 5'-tetraphosphate
3.6.1.9
3.6.1.8
3.6.1.5
3.6.1.15
2.7.4.3
2.7.4.10
4.6.1.1
3',5'-Cyclic AMP
1.17.4.1
1.17.4.2
1.1.98.6
dADP
dATP
2.7.4.11
dAMP
Deoxyadenosine
3.1.3.89
2.7.1.76
2.7.1.145
3.5.4.4
Deoxyinosine
Urate-3-ribonucleoside
2.4.2.16
Urate
1.14.13.113
1.7.3.3
5-Hydroxyisourate
3.5.2.17
5-Hydroxy-2-oxo-4-ureido-2,5-dihydro-1H-imidazole-5-carboxylate
4.1.1.97
(S)-Allantoin
3.5.2.5
spontaneous
5.1.99.3
(R)-Allantoin
3.5.2.-
5-Ureido-4-imidazole carboxylate
3.5.3.-
5-Amino-4-imidazole carboxylate
4.1.1.-
Amino-imidazole
3.5.4.8
Imidazolone
3.5.2.-
Formiminoglycine
2.1.2.4
Glycine
Glycine, serine and threonine metabolism
Ureidoglycine
2.6.1.112
3.5.3.9
3.5.3.26
2.1.3.16
Allantoate
3.5.3.4
Ureidoglycolate
1.1.1.154
1.1.1.350
Oxalureate
2.1.3.5
Carbamoyl phosphate
2.7.2.2
Hydrogen carbonate
Oxamate
3.5.1.126
4.3.2.3
3.5.1.116
Urea
3.5.1.5
Glyoxylate
Glyoxylate metabolism
NH3
CO2
Oxalate
00230 6/27/22
(c) Kanehisa Laboratories

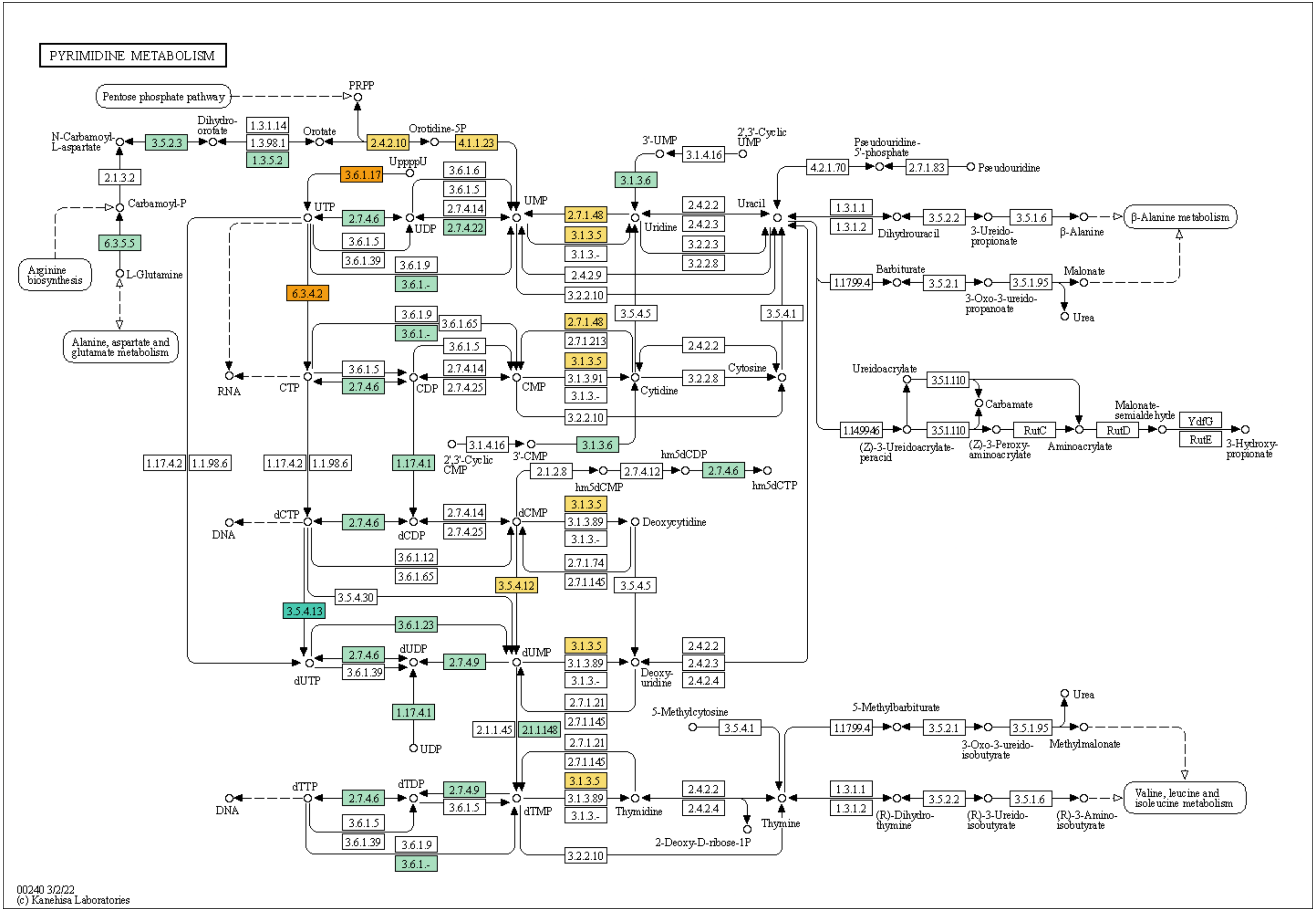

PYRIMIDINE METABOLISM
Pentose phosphate pathway
PRPP
N-Carbamoyl-L-aspartate
3.5.2.3
Dihydro-orotate
1.3.1.14
1.3.98.1
1.3.5.2
Orotate
2.4.2.10
Orotidine-5P
4.1.1.23
2.1.3.2
Carbamoyl-P
6.3.5.5
L-Glutamine
Arginine biosynthesis
Alanine, aspartate and glutamate metabolism
3.6.1.17
UppppU
3.6.1.6
3.6.1.5
2.7.4.14
2.7.4.22
UTP
2.7.4.6
UDP
UMP
3.6.1.5
3.6.1.39
3.6.1.9
3.6.1.-
6.3.4.2
3'-UMP
3.1.4.16
2',3'-Cyclic UMP
3.1.3.6
2.7.1.48
3.1.3.5
3.1.3.-
Uridine
2.4.2.9
3.2.2.10
2.4.2.2
2.4.2.3
3.2.2.3
3.2.2.8
Uracil
4.2.1.70
Pseudouridine-5'-phosphate
2.7.1.83
Pseudouridine
1.3.1.1
1.3.1.2
Dihydrouracil
3.5.2.2
3-Ureido-propionate
3.5.1.6
β-Alanine
β-Alanine metabolism
1.17.99.4
Barbiturate
3.5.2.1
3-Oxo-3-ureido-propanoate
3.5.1.95
Malonate
Urea
3.6.1.9
3.6.1.65
3.6.1.-
3.6.1.5
3.6.1.5
2.7.4.6
2.7.4.14
2.7.4.25
RNA
CTP
CDP
CMP
2.7.1.48
2.7.1.213
3.1.3.5
3.1.3.91
3.1.3.-
3.2.2.10
3.5.4.5
3.5.4.1
2.4.2.2
3.2.2.8
Cytidine
Cytosine
Ureidoacrylate
3.5.1.110
Carbamate
1.14.99.46
(Z)-3-Ureidoacrylate-peracid
3.5.1.110
(Z)-3-Peroxy-aminoacrylate
RutC
Aminoacrylate
RutD
Malonate-semialdehyde
YdfG
RutE
3-Hydroxy-propionate
3.1.4.16
2',3'-Cyclic CMP
3'-CMP
3.1.3.6
1.17.4.2
1.1.98.6
1.17.4.2
1.1.98.6
1.17.4.1
2.1.2.8
hm5dCMP
2.7.4.12
hm5dCDP
2.7.4.6
hm5dCTP
DNA
dCTP
2.7.4.6
dCDP
2.7.4.14
2.7.4.25
dCMP
3.1.3.5
3.1.3.89
3.1.3.-
Deoxycytidine
3.6.1.12
3.6.1.65
2.7.1.74
2.7.1.145
3.5.4.12
3.5.4.5
3.5.4.30
3.5.4.13
3.6.1.23
dUTP
2.7.4.6
3.6.1.39
dUDP
2.7.4.9
dUMP
3.1.3.5
3.1.3.89
3.1.3.-
Deoxy-uridine
2.4.2.2
2.4.2.3
2.4.2.4
1.17.4.1
UDP
2.1.1.45
2.1.1.148
2.7.1.21
2.7.1.145
2.7.1.21
2.7.1.145
5-Methylcytosine
3.5.4.1
5-Methylbarbiturate
1.17.99.4
3.5.2.1
3-Oxo-3-ureido-isobutyrate
3.5.1.95
Methylmalonate
Urea
DNA
dTTP
2.7.4.6
dTDP
2.7.4.9
3.6.1.5
dTMP
3.1.3.5
3.1.3.89
3.1.3.-
Thymidine
2.4.2.2
2.4.2.4
2-Deoxy-D-ribose-1P
Thymine
1.3.1.1
1.3.1.2
(R)-Dihydro-thymine
3.5.2.2
(R)-3-Ureido-isobutyrate
3.5.1.6
(R)-3-Amino-isobutyrate
Valine, leucine and isoleucine metabolism
3.6.1.5
3.6.1.39
3.6.1.9
3.6.1.-
3.2.2.10
00240 3/2/22
(c) Kanehisa Laboratories

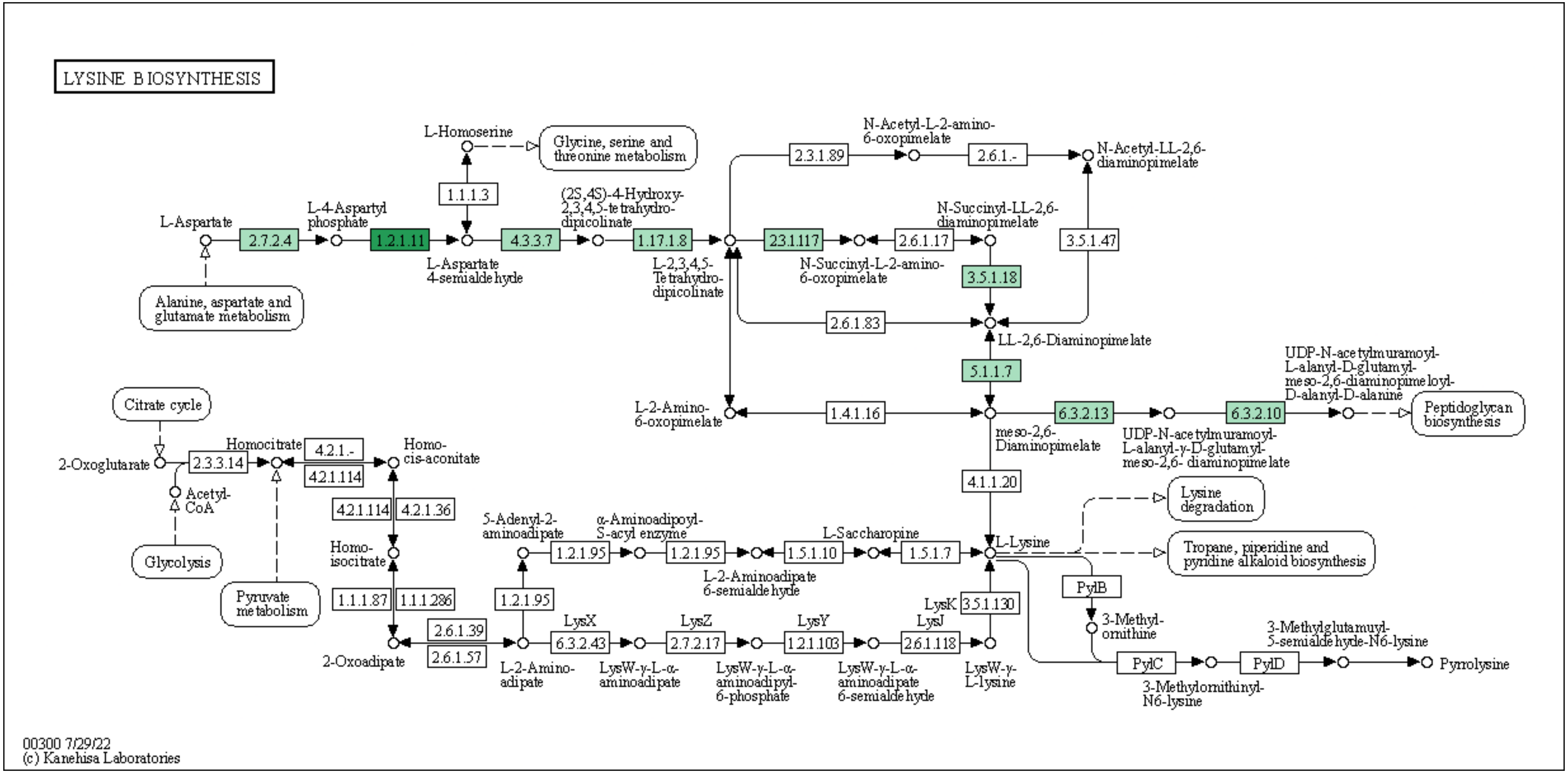
LYSINE BIOSYNTHESIS
L-Homoserine
Glycine, serine and threonine metabolism
1.1.1.3
L-Aspartate
2.7.2.4
L-4-Aspartyl phosphate
1.2.1.11
L-Aspartate 4-semialdehyde
4.3.3.7
(2S,4S)-4-Hydroxy-2,3,4,5-tetrahydro-dipicolinate
1.17.1.8
L-2,3,4,5-Tetrahydro-dipicolinate
Alanine, aspartate and glutamate metabolism
2.3.1.89
N-Acetyl-L-2-amino-6-oxopimelate
2.6.1.-
N-Acetyl-LL-2,6-diaminopimelate
2.3.1.117
2.6.1.17
N-Succinyl-LL-2,6-diaminopimelate
N-Succinyl-L-2-amino-6-oxopimelate
3.5.1.47
3.5.1.18
2.6.1.83
LL-2,6-Diaminopimelate
5.1.1.7
L-2-Amino-6-oxopimelate
1.4.1.16
meso-2,6-Diaminopimelate
6.3.2.13
UDP-N-acetylmuramoyl-L-alanyl-D-glutamyl-meso-2,6-diaminopimelate
6.3.2.10
UDP-N-acetylmuramoyl-L-alanyl-D-glutamyl-meso-2,6-diaminopimeloyl-D-alanyl-D-alanine
Peptidoglycan biosynthesis
Citrate cycle
2-Oxoglutarate
2.3.3.14
Homocitrate
4.2.1.-
4.2.1.114
Homo-cis-aconitate
Acetyl-CoA
Glycolysis
Pyruvate metabolism
4.2.1.114
4.2.1.36
Homo-isocitrate
1.1.1.87
1.1.1.286
2-Oxoadipate
2.6.1.39
2.6.1.57
5-Adenyl-2-aminoadipate
1.2.1.95
α-Aminoadipoyl-S-acyl enzyme
1.2.1.95
L-2-Aminoadipate 6-semialdehyde
1.5.1.10
L-Saccharopine
1.5.1.7
4.1.1.20
L-Lysine
Lysine degradation
Tropane, piperidine and pyridine alkaloid biosynthesis
1.2.1.95
L-2-Amino-adipate
LysX
6.3.2.43
LysW-γ-L-α-aminoadipate
LysZ
2.7.2.17
LysW-γ-L-α-aminoadipyl-6-phosphate
LysY
1.2.1.103
LysW-γ-L-α-aminoadipate 6-semialdehyde
LysJ
2.6.1.118
LysK
3.5.1.130
LysW-γ-L-lysine
PylB
3-Methyl-ornithine
PylC
3-Methylornithinyl-N6-lysine
PylD
3-Methylglutamyl-5-semialdehyde-N6-lysine
Pyrrolysine
00300 7/29/22
(c) Kanehisa Laboratories

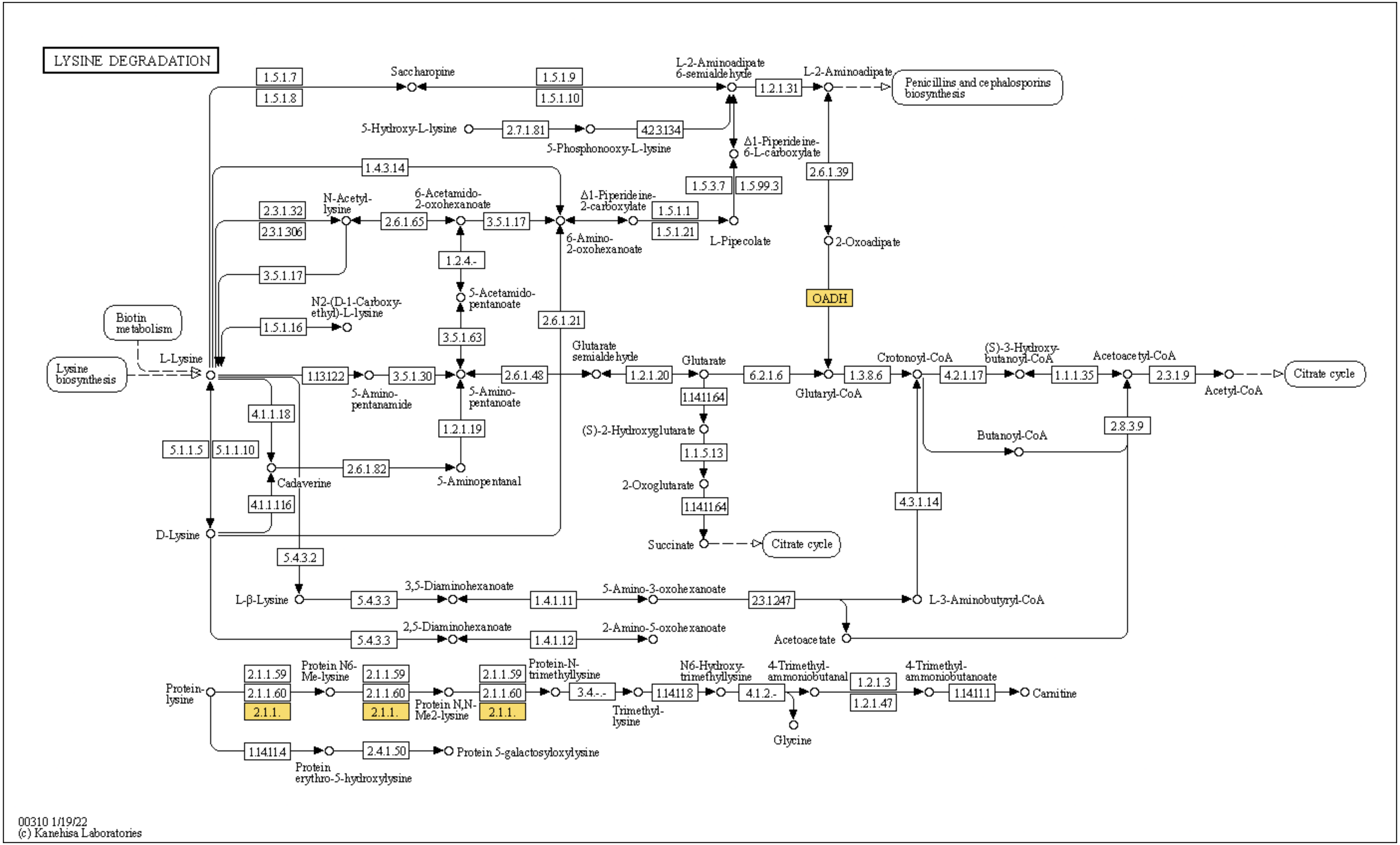
LYSINE DEGRADATION
1.5.1.7
1.5.1.8
Saccharopine
1.5.1.9
1.5.1.10
L-2-Aminoadipate 6-semialdehyde
1.2.1.31
L-2-Aminoadipate
Penicillins and cephalosporins biosynthesis
5-Hydroxy-L-lysine
2.7.1.81
4.2.3.134
5-Phosphonooxy-L-lysine
Δ1-Piperideine-6-L-carboxylate
1.4.3.14
1.5.3.7
1.5.99.3
2.6.1.39
2.3.1.32
2.3.1.306
N-Acetyl-lysine
6-Acetamido-2-oxohexanoate
2.6.1.65
3.5.1.17
Δ1-Piperideine-2-carboxylate
1.5.1.1
1.5.1.21
L-Pipecolate
6-Amino-2-oxohexanoate
2-Oxoadipate
3.5.1.17
1.2.4.-
5-Acetamido-pentanoate
N2-(D-1-Carboxy-ethyl)-L-lysine
1.5.1.16
OADH
Biotin metabolism
3.5.1.63
2.6.1.21
Glutarate semialdehyde
Glutarate
Crotonoyl-CoA
(S)-3-Hydroxy-butanoyl-CoA
Acetoacetyl-CoA
Lysine biosynthesis
L-Lysine
1.13.12.2
3.5.1.30
2.6.1.48
1.2.1.20
6.2.1.6
1.3.8.6
4.2.1.17
1.1.1.35
2.3.1.9
Citrate cycle
5-Amino-pentanamide
5-Amino-pentanoate
Glutaryl-CoA
Acetyl-CoA
1.14.11.64
4.1.1.18
(S)-2-Hydroxyglutarate
Butanoyl-CoA
2.8.3.9
1.2.1.19
5.1.1.5
5.1.1.10
2.6.1.82
1.1.5.13
Cadaverine
5-Aminopentanal
2-Oxoglutarate
4.1.1.116
4.3.1.14
1.14.11.64
D-Lysine
Succinate
Citrate cycle
5.4.3.2
3,5-Diaminohexanoate
5-Amino-3-oxohexanoate
L-β-Lysine
5.4.3.3
1.4.1.11
2.3.1.247
L-3-Aminobutyryl-CoA
Acetoacetate
2,5-Diaminohexanoate
2-Amino-5-oxohexanoate
5.4.3.3
1.4.1.12
Protein-lysine
2.1.1.59
2.1.1.60
2.1.1.
Protein N6-Me-lysine
2.1.1.59
2.1.1.60
2.1.1.
Protein N,N-Me2-lysine
2.1.1.59
2.1.1.60
2.1.1.
Protein-N-trimethyllysine
3.4.-.-
Trimethyl-lysine
N6-Hydroxy-trimethyllysine
1.14.11.8
4-Trimethyl-ammoniobutanal
4.1.2.-
Glycine
1.2.1.3
1.2.1.47
4-Trimethyl-ammoniobutanoate
1.14.11.1
Carnitine
1.14.11.4
Protein erythro-5-hydroxylysine
2.4.1.50
Protein 5-galactosyloxylysine
00310 1/19/22
(c) Kanehisa Laboratories

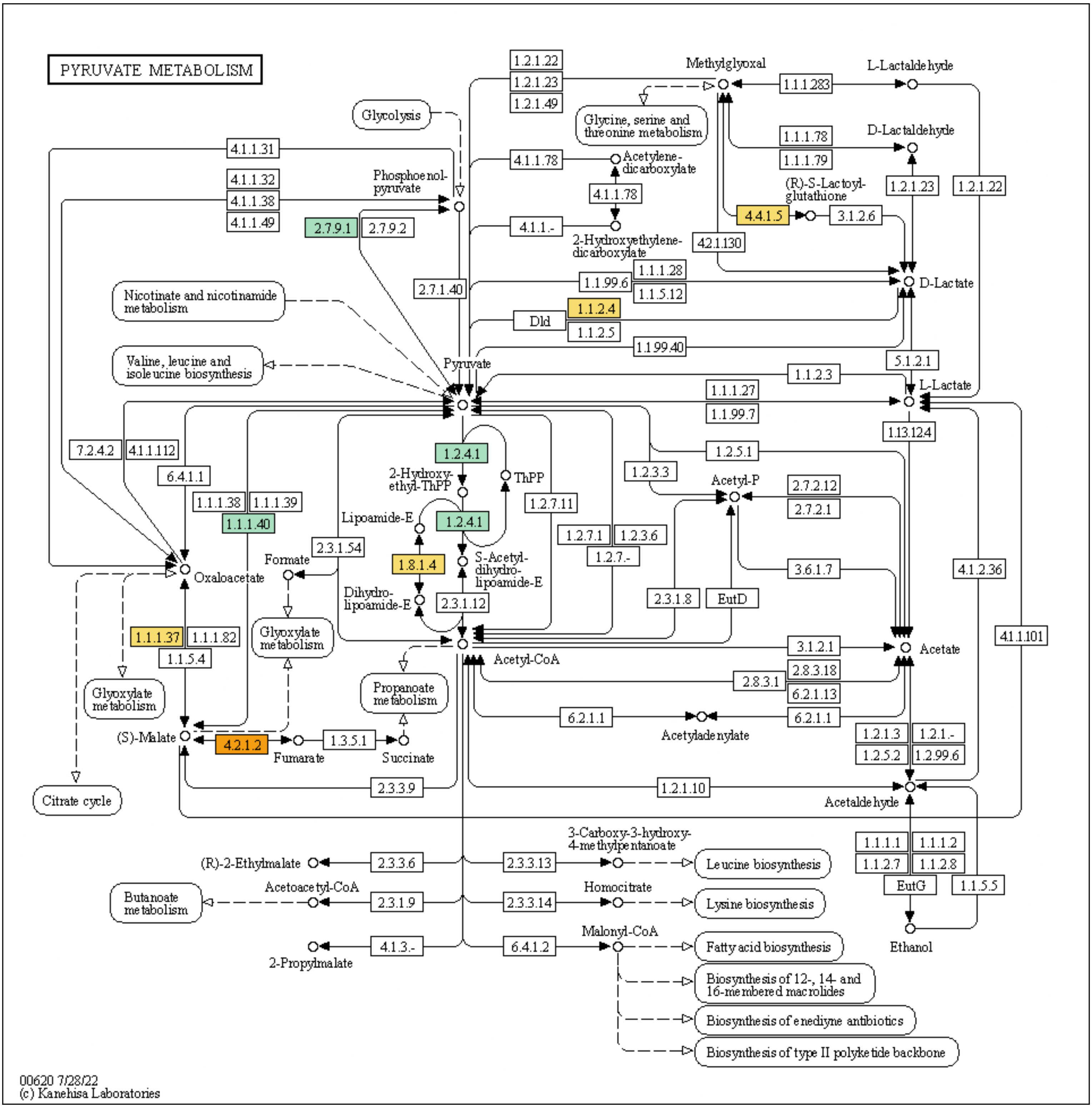
PYRUVATE METABOLISM
Glycolysis
Phosphoenol-pyruvate
Pyruvate
Oxaloacetate
(S)-Malate
Fumarate
Succinate
Formate
Acetyl-CoA
Methylglyoxal
L-Lactaldehyde
D-Lactaldehyde
(R)-S-Lactoyl-glutathione
D-Lactate
L-Lactate
Acetyl-P
Acetate
Acetyladenylate
Acetaldehyde
Ethanol
Acetylene-dicarboxylate
2-Hydroxyethylene-dicarboxylate
2-Hydroxy-ethyl-ThPP
ThPP
Lipoamide-E
S-Acetyl-dihydro-lipoamide-E
Dihydro-lipoamide-E
(R)-2-Ethylmalate
Acetoacetyl-CoA
2-Propylmalate
3-Carboxy-3-hydroxy-4-methylpentanoate
Homocitrate
Malonyl-CoA
Glycine, serine and threonine metabolism
Nicotinate and nicotinamide metabolism
Valine, leucine and isoleucine biosynthesis
Glyoxylate metabolism
Propanoate metabolism
Citrate cycle
Butanoate metabolism
Leucine biosynthesis
Lysine biosynthesis
Fatty acid biosynthesis
Biosynthesis of 12-, 14- and 16-membered macrolides
Biosynthesis of enediyne antibiotics
Biosynthesis of type II polyketide backbone
1.2.1.22
1.2.1.23
1.2.1.49
1.1.1.283
1.1.1.78
1.1.1.79
1.2.1.23
1.2.1.22
4.4.1.5
3.1.2.6
4.2.1.130
4.1.1.31
4.1.1.32
4.1.1.38
4.1.1.49
2.7.9.1
2.7.9.2
4.1.1.78
4.1.1.78
4.1.1.-
2.7.1.40
1.1.1.28
1.1.99.6
1.1.5.12
Dld
1.1.2.4
1.1.2.5
1.1.99.40
5.1.2.1
1.1.2.3
1.1.1.27
1.1.99.7
1.13.12.4
7.2.4.2
4.1.1.112
6.4.1.1
1.2.4.1
1.2.4.1
1.1.1.38
1.1.1.39
1.1.1.40
2.3.1.54
1.8.1.4
2.3.1.12
1.2.7.11
1.2.7.1
1.2.3.6
1.2.7.-
1.2.3.3
1.2.5.1
2.7.2.12
2.7.2.1
3.6.1.7
2.3.1.8
EutD
4.1.2.36
4.1.1.101
1.1.1.37
1.1.1.82
1.1.5.4
3.1.2.1
2.8.3.18
2.8.3.1
6.2.1.13
6.2.1.1
6.2.1.1
4.2.1.2
1.3.5.1
1.2.1.3
1.2.1.-
1.2.5.2
1.2.99.6
2.3.3.9
1.2.1.10
1.1.1.1
1.1.1.2
1.1.2.7
1.1.2.8
EutG
1.1.5.5
2.3.3.6
2.3.3.13
2.3.1.9
2.3.3.14
4.1.3.-
6.4.1.2
00620 7/28/22
(c) Kanehisa Laboratories

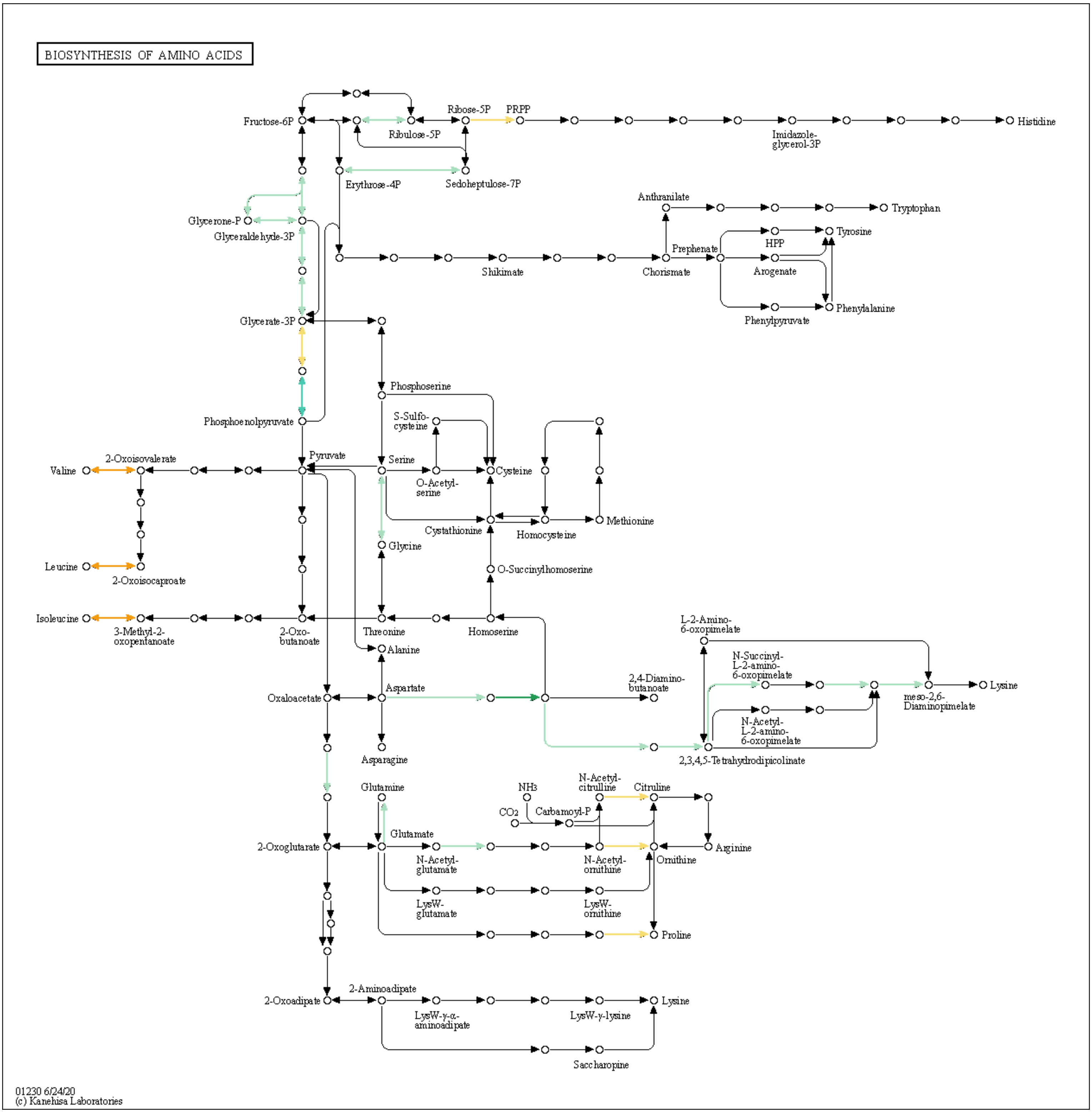
BIOSYNTHESIS OF AMINO ACIDS
Fructose-6P
Ribose-5P
PRPP
Histidine
Ribulose-5P
Imidazole-glycerol-3P
Erythrose-4P
Sedoheptulose-7P
Anthranilate
Tryptophan
Glycerone-P
Glyceraldehyde-3P
Tyrosine
HPP
Prephenate
Shikimate
Chorismate
Arogenate
Phenylalanine
Phenylpyruvate
Glycerate-3P
Phosphoserine
S-Sulfo-cysteine
Phosphoenolpyruvate
2-Oxoisovalerate
Pyruvate
Serine
Valine
Cysteine
O-Acetyl-serine
Methionine
Cystathionine
Homocysteine
Glycine
Leucine
2-Oxoisocaproate
O-Succinylhomoserine
Isoleucine
3-Methyl-2-oxopentanoate
2-Oxo-butanoate
Threonine
Homoserine
L-2-Amino-6-oxopimelate
Alanine
N-Succinyl-L-2-amino-6-oxopimelate
2,4-Diamino-butanoate
Aspartate
Oxaloacetate
Lysine
meso-2,6-Diaminopimelate
N-Acetyl-L-2-amino-6-oxopimelate
Asparagine
2,3,4,5-Tetrahydrodipicolinate
Glutamine
NH3
N-Acetyl-citrulline
Citrulline
CO2
Carbamoyl-P
Glutamate
2-Oxoglutarate
Arginine
N-Acetyl-glutamate
N-Acetyl-ornithine
Ornithine
LysW-glutamate
LysW-ornithine
Proline
2-Aminoadipate
2-Oxoadipate
Lysine
LysW-γ-α-aminoadipate
LysW-γ-lysine
Saccharopine
01230 6/24/20
(c) Kanehisa Laboratories

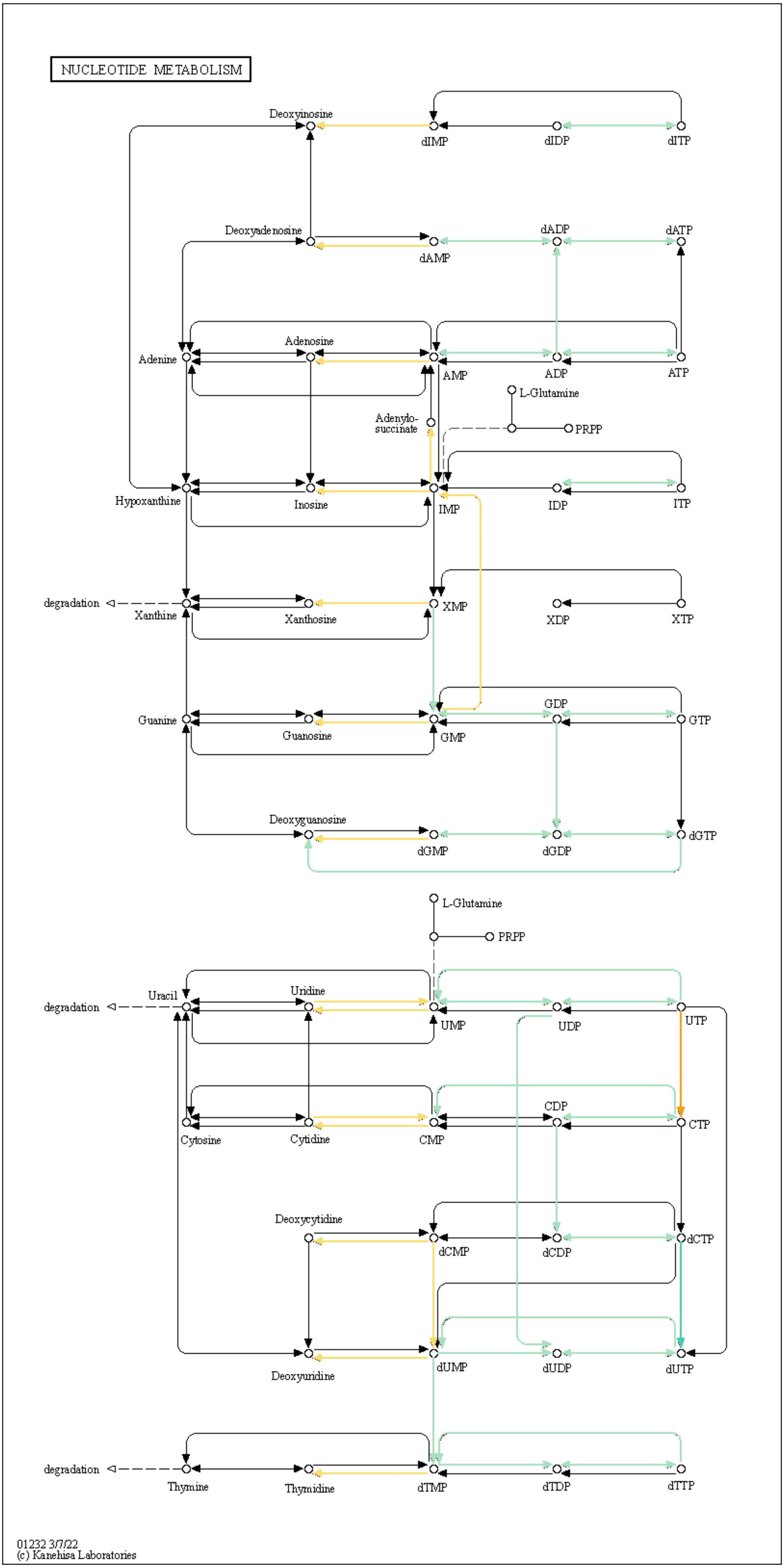
NUCLEOTIDE METABOLISM
Deoxyinosine
dIMP
dIDP
dITP
Deoxyadenosine
dAMP
dADP
dATP
Adenosine
Adenine
AMP
ADP
ATP
L-Glutamine
Adenylo-
succinate
PRPP
Hypoxanthine
Inosine
IMP
IDP
ITP
degradation
Xanthine
Xanthosine
XMP
XDP
XTP
GDP
Guanine
Guanosine
GMP
GTP
Deoxyguanosine
dGMP
dGDP
dGTP
L-Glutamine
PRPP
Uracil
Uridine
degradation
UMP
UDP
UTP
CDP
Cytosine
Cytidine
CMP
CTP
Deoxycytidine
dCMP
dCDP
dCTP
Deoxyuridine
dUMP
dUDP
dUTP
degradation
Thymine
Thymidine
dTMP
dTDP
dTTP
01232 3/7/22
(c) Kanehisa Laboratories

RNA POLYMERASE

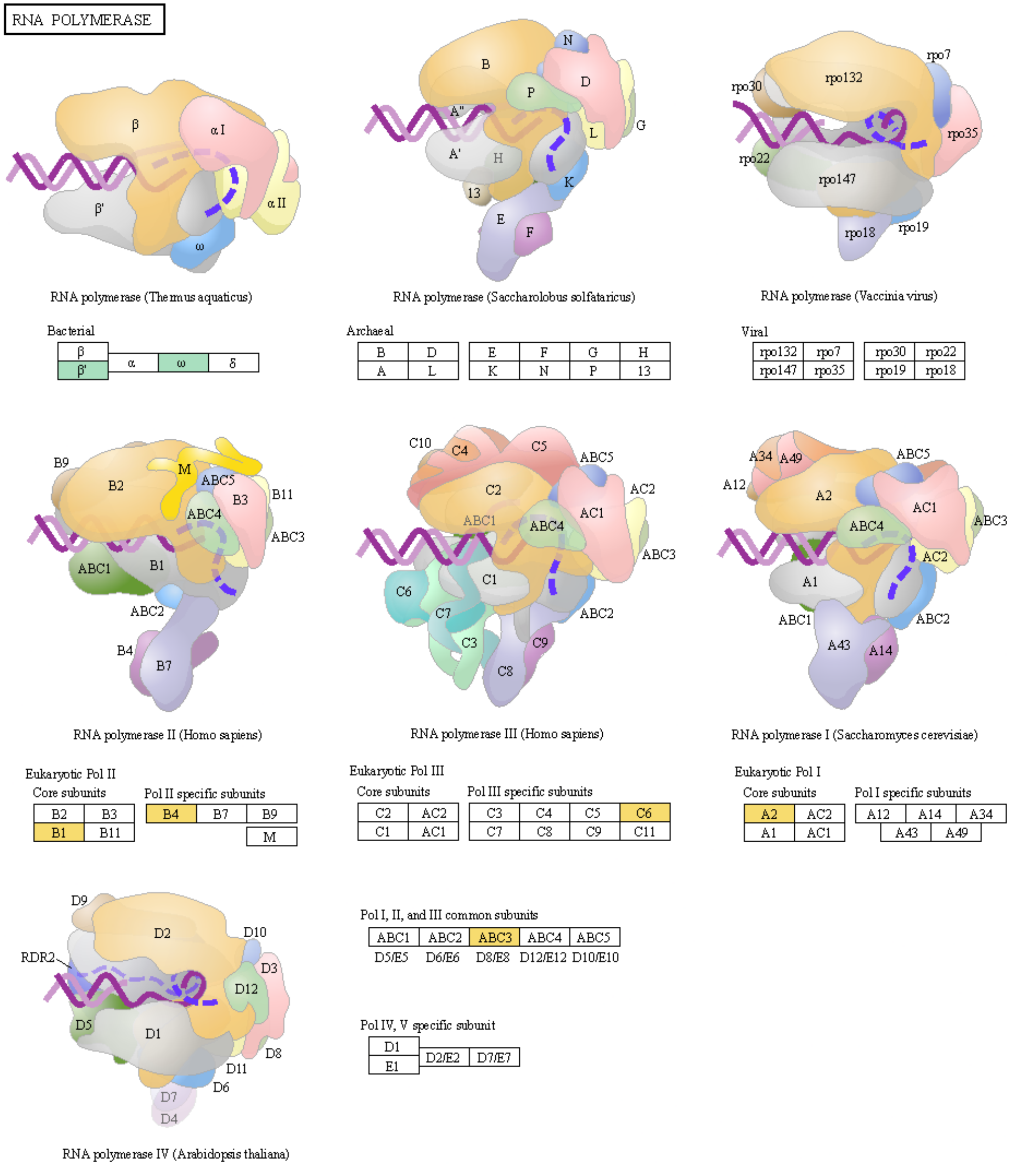

03020 3/14/22

(c) Kanehisa Laboratories

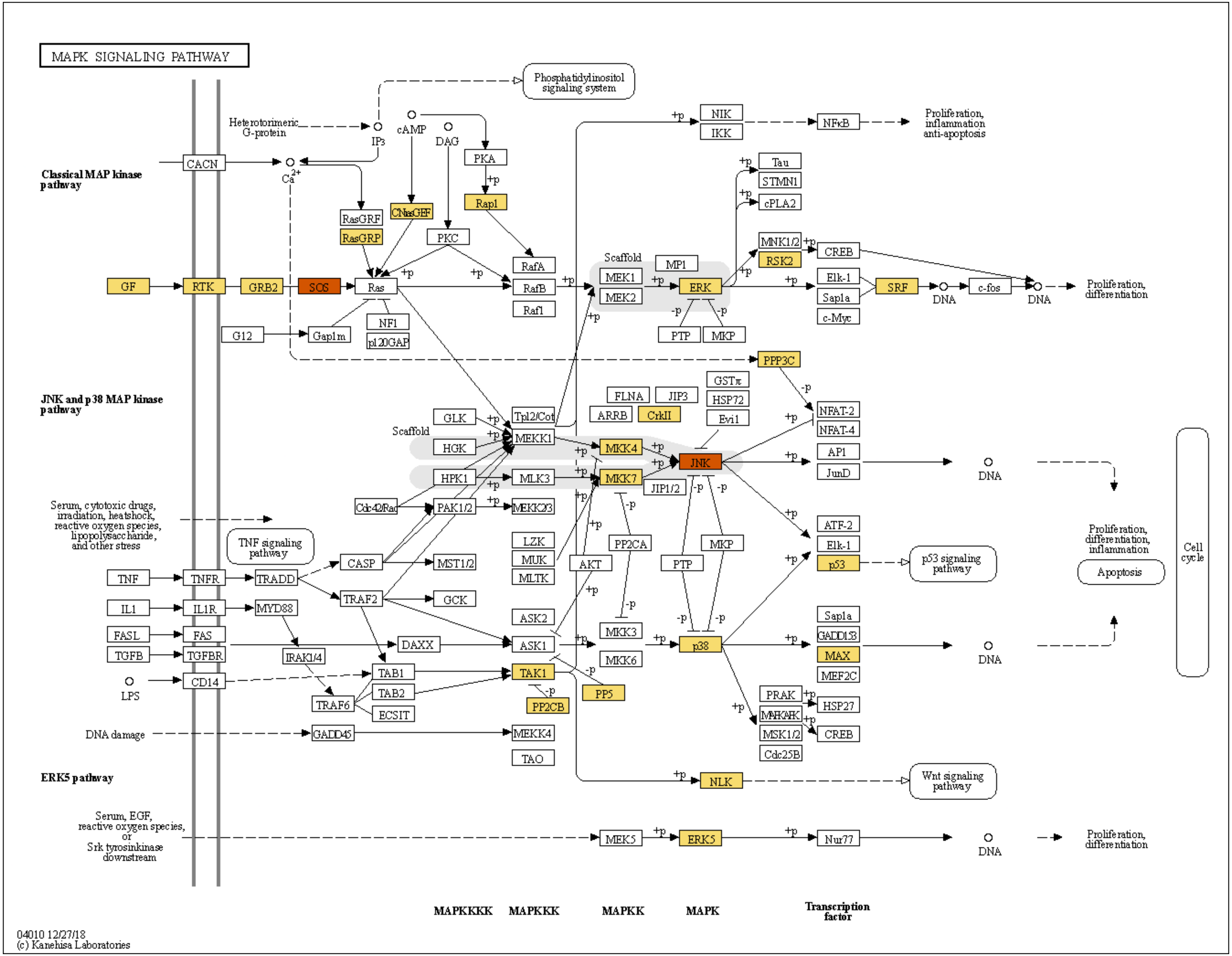

MAPK SIGNALING PATHWAY
Classical MAP kinase pathway
JNK and p38 MAP kinase pathway
ERK5 pathway
Phosphatidylinositol signaling system
Heterotorimeric G-protein
IP3
cAMP
DAG
CACN
Ca2+
PKA
Rap1
RasGRF
RasGRP
CNrasGEF
PKC
GF
RTK
GRB2
SOS
Ras
RafA
RafB
Raf1
NF1
p120GAP
G12
Gap1m
Scaffold
MEK1
MEK2
MP1
ERK
PTP
MKP
NIK
IKK
NFκB
Proliferation, inflammation anti-apoptosis
Tau
STMN1
cPLA2
MNK1/2
RSK2
CREB
Elk-1
Sap1a
c-Myc
SRF
c-fos
DNA
Proliferation, differentiation
PPP3C
NFAT-2
NFAT-4
FLNA
JIP3
ARRB
CrkII
GSTπ
HSP72
Evi1
GLK
HGK
HPK1
Tpl2/Cot
MEKK1
MLK3
MEKK2/3
MKK4
MKK7
JNK
JIP1/2
AP1
JunD
Cdc42/Rac
PAK1/2
Serum, cytotoxic drugs, irradiation, heatshock, reactive oxygen species, lipopolysaccharide, and other stress
TNF signaling pathway
LZK
MUK
MLTK
AKT
PP2CA
ATF-2
p53
p53 signaling pathway
Proliferation, differentiation, inflammation
Apoptosis
Cell cycle
CASP
MST1/2
TNF
TNFR
TRADD
TRAF2
GCK
IL1
IL1R
MYD88
FASL
FAS
DAXX
ASK2
ASK1
MKK3
MKK6
p38
Sap1a
GADD153
MAX
MEF2C
TGFB
TGFBR
IRAK1/4
LPS
CD14
TAB1
TAB2
TRAF6
ECSIT
TAK1
PP2CB
PP5
PRAK
MAPKAPK
MSK1/2
Cdc25B
HSP27
DNA damage
GADD45
MEKK4
TAO
NLK
Wnt signaling pathway
Serum, EGF, reactive oxygen species, or Srk tyrosinkinase downstream
MEK5
ERK5
Nur77
MAPKKKK
MAPKKK
MAPKK
MAPK
Transcription factor
04010 12/27/18
(c) Kanehisa Laboratories

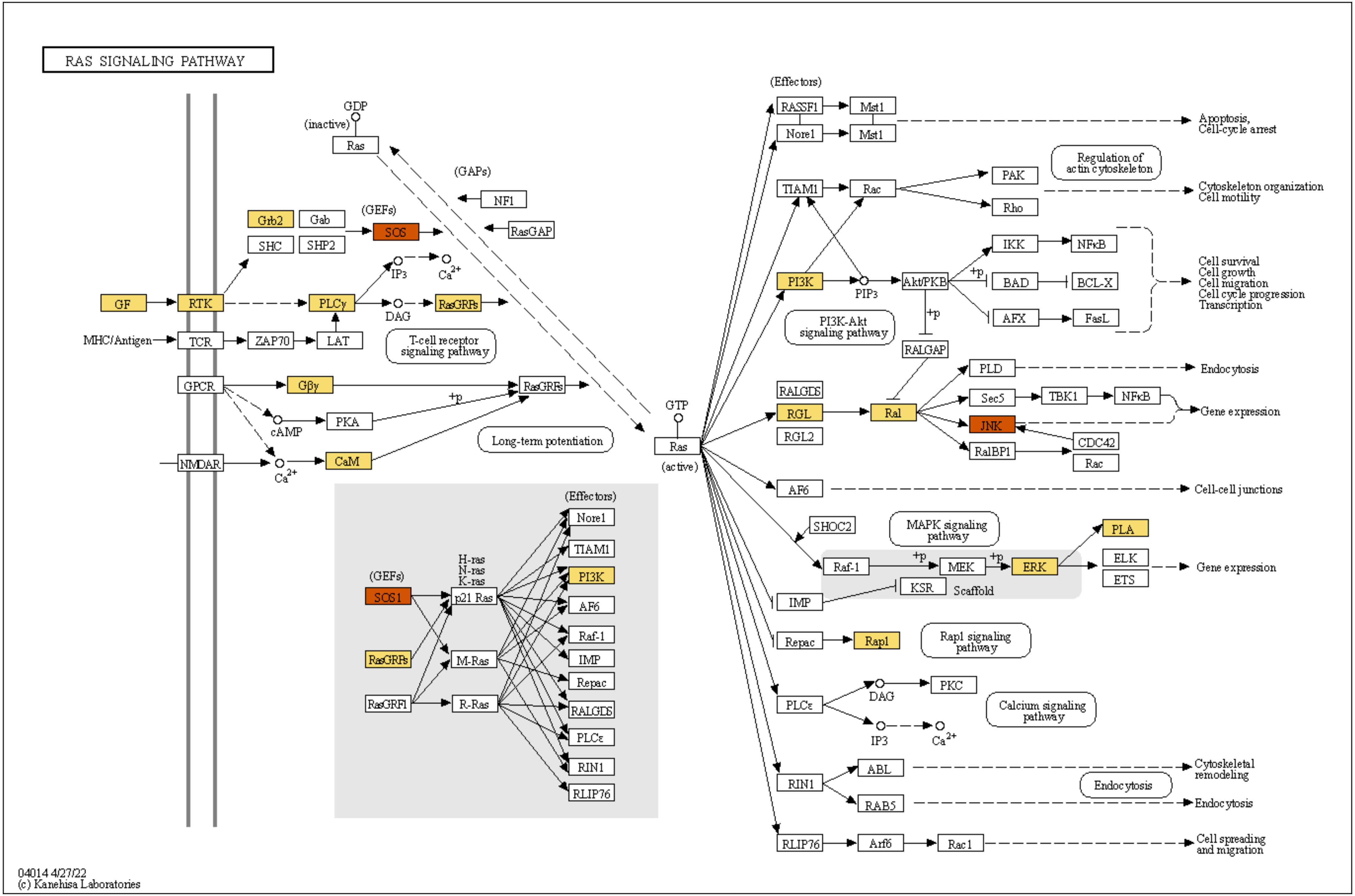
RAS SIGNALING PATHWAY
GDP
(inactive)
Ras
(GAPs)
NF1
RasGAP
(GEFs)
Grb2
Gab
SOS
SHC
SHP2
IP3
Ca2+
GF
RTK
PLCγ
DAG
RasGRFs
MHC/Antigen
TCR
ZAP70
LAT
T-cell receptor signaling pathway
GPCR
Gβγ
RasGRFs
+p
cAMP
PKA
Long-term potentiation
NMDAR
Ca2+
CaM
GTP
Ras
(active)
(Effectors)
Nore1
TIAM1
PI3K
AF6
Raf-1
IMP
Repac
RALGDS
PLCε
RIN1
RLIP76
H-ras
N-ras
K-ras
(GEFs)
SOS1
p21 Ras
RasGRFs
M-Ras
RasGRF1
R-Ras
(Effectors)
RASSF1
Mst1
Nore1
Mst1
Apoptosis, Cell-cycle arrest
Regulation of actin cytoskeleton
TIAM1
Rac
PAK
Rho
Cytoskeleton organization
Cell motility
IKK
NFκB
PI3K
PIP3
Akt/PKB
+p
BAD
BCL-X
AFX
FasL
Cell survival
Cell growth
Cell migration
Cell cycle progression
Transcription
PI3K-Akt signaling pathway
+p
RALGAP
PLD
Endocytosis
RALGDS
Sec5
TBK1
NFκB
RGL
Ral
Gene expression
JNK
RGL2
CDC42
RalBP1
Rac
AF6
Cell-cell junctions
SHOC2
MAPK signaling pathway
PLA
Raf-1
+p
MEK
+p
ERK
ELK
ETS
Gene expression
KSR
Scaffold
IMP
Repac
Rap1
Rap1 signaling pathway
DAG
PKC
PLCε
Calcium signaling pathway
IP3
Ca2+
ABL
Cytoskeletal remodeling
RIN1
Endocytosis
RAB5
Endocytosis
RLIP76
Arf6
Rac1
Cell spreading and migration
04014 4/27/22
(c) Kanehisa Laboratories

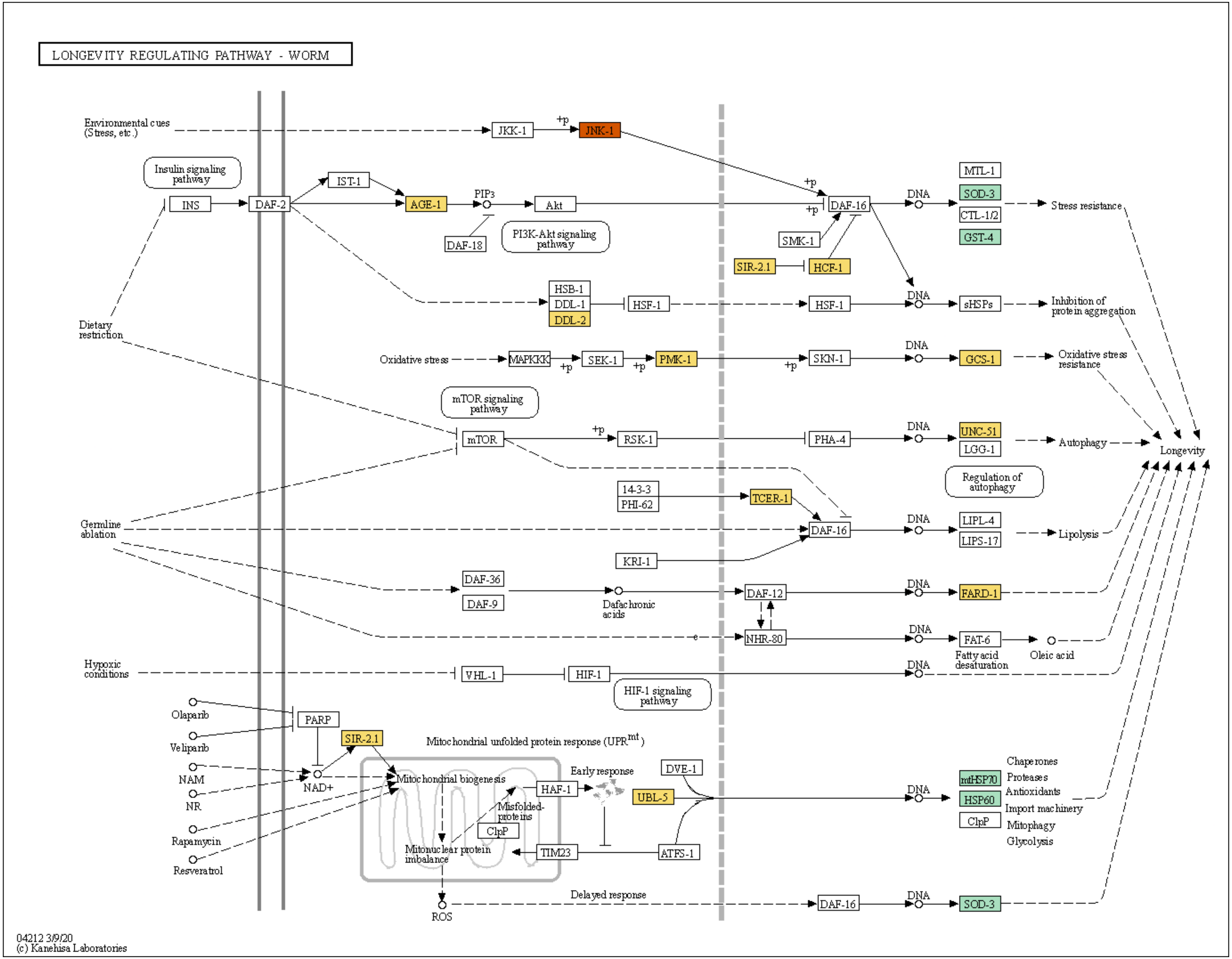
LONGEVITY REGULATING PATHWAY - WORM
Environmental cues (Stress, etc.)
JKK-1
+p
JNK-1
Insulin signaling pathway
INS
DAF-2
IST-1
AGE-1
PIP3
Akt
DAF-18
PI3K-Akt signaling pathway
+p
DAF-16
SMK-1
SIR-2.1
HCF-1
DNA
MTL-1
SOD-3
CTL-1/2
GST-4
Stress resistance
HSB-1
DDL-1
DDL-2
HSF-1
HSF-1
sHSPs
Inhibition of protein aggregation
Dietary restriction
Oxidative stress
MAPKKK
SEK-1
PMK-1
SKN-1
GCS-1
Oxidative stress resistance
mTOR signaling pathway
mTOR
RSK-1
PHA-4
UNC-51
LGG-1
Autophagy
Longevity
Regulation of autophagy
14-3-3
PHI-62
TCER-1
Germline ablation
DAF-16
LIPL-4
LIPS-17
Lipolysis
KRI-1
DAF-36
DAF-9
Dafachronic acids
DAF-12
FARD-1
NHR-80
FAT-6
Fatty acid desaturation
Oleic acid
Hypoxic conditions
VHL-1
HIF-1
HIF-1 signaling pathway
Olaparib
PARP
Veliparib
SIR-2.1
NAM
NAD+
NR
Rapamycin
Resveratrol
Mitochondrial unfolded protein response (UPRmt)
Mitochondrial biogenesis
HAF-1
Early response
DVE-1
UBL-5
Misfolded-proteins
ClpP
Mitonuclear protein imbalance
TIM23
ATFS-1
mtHSP70
HSP60
ClpP
Chaperones
Proteases
Antioxidants
Import machinery
Mitophagy
Glycolysis
ROS
Delayed response
DAF-16
SOD-3
04212 3/9/20
(c) Kanehisa Laboratories

OXIDATIVE PHOSPHORYLATION

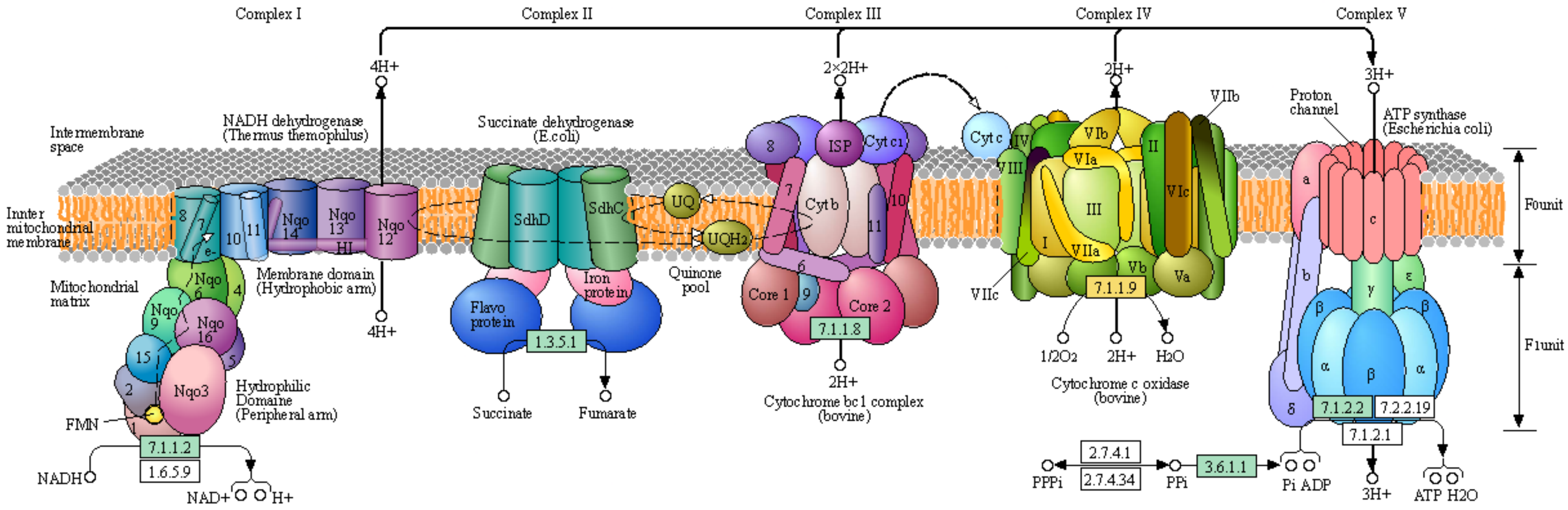

NADH dehydrogenase

E	ND1	ND2	ND3	ND4	ND4L	ND5	ND6										
E	Ndufs1	Ndufs2	Ndufs3	Ndufs4	Ndufs5	Ndufs6	Ndufs7	Ndufs8	Ndufv1	Ndufv2	Ndufv3						
B/A	NuoA	NuoB	NuoC	NuoD	NuoE	NuoF	NuoG	NuoH	NuoI	NuoJ	NuoK	NuoL	NuoM	NuoN			
B/A	NdhC	NdhK	NdhJ	NdhH	NdhA	NdhI	NdhG	NdhE	NdhF	NdhD	NdhB	NdhL	NdhM	NdhN	HoxE	HoxF	HoxU
E	Ndufa1	Ndufa2	Ndufa3	Ndufa4	Ndufa5	Ndufa6	Ndufa7	Ndufa8	Ndufa9	Ndufa10	Ndufab1	Ndufa11	Ndufa12	Ndufa13			
E	Ndufb1	Ndufb2	Ndufb3	Ndufb4	Ndufb5	Ndufb6	Ndufb7	Ndufb8	Ndufb9	Ndufb10	Ndufb11	Ndufc1	Ndufc2				

Succinate dehydrogenase / Fumarate reductase

E	SDHC	SDHD	SDHA	SDHB		
B/A	SdhC	SdhD	SdhA	SdhB		
			FrdA	FrdB	FrdC	FrdD

Cytochrome c reductase

E/B/A	ISP	Cytb	Cyt1							
E				COR1	QCR2	QCR6	QCR7	QCR8	QCR9	QCR10

Cytochrome c oxidase

E	COX10		COX3	COX1	COX2	COX4	COX5A	COX5B	COX6A	COX6B	COX6C	COX7A	COX7B	COX7C	COX8
B/A	CyoE	CyoD	CyoC	CyoB	CyoA										
		CoxD	CoxC	CoxA	CoxB										
		QoxD	QoxC	QoxB	QoxA										
		SoxD	SoxC	SoxB	SoxA										

E/B/A	COX11	COX15	
			COX17

Cytochrome c oxidase , cbb3-type

B	I	II	IV	III

Cytochrome bd complex

B/A	CydA	CydB	CydX

Cytochrome c

CYC

F-type ATPase (Bacteria)

alpha	beta	gamma	delta	epsilon
a	b	c		

F-type ATPase (Eukaryotes)

alpha	beta	gamma	delta	epsilon	
OSCP	a	b	c	d	e
f	g	f6/h	j	k	8

V/A-type ATPase (Bacteria, Archaea)

A	B	C	D	E	F	G/H
I	K					

V-type ATPase (Eukaryotes)

A	B	C	D	E	F	G	H
a	c	d	e	S1			

00190 7/28/22
(c) Kanehisa Laboratories

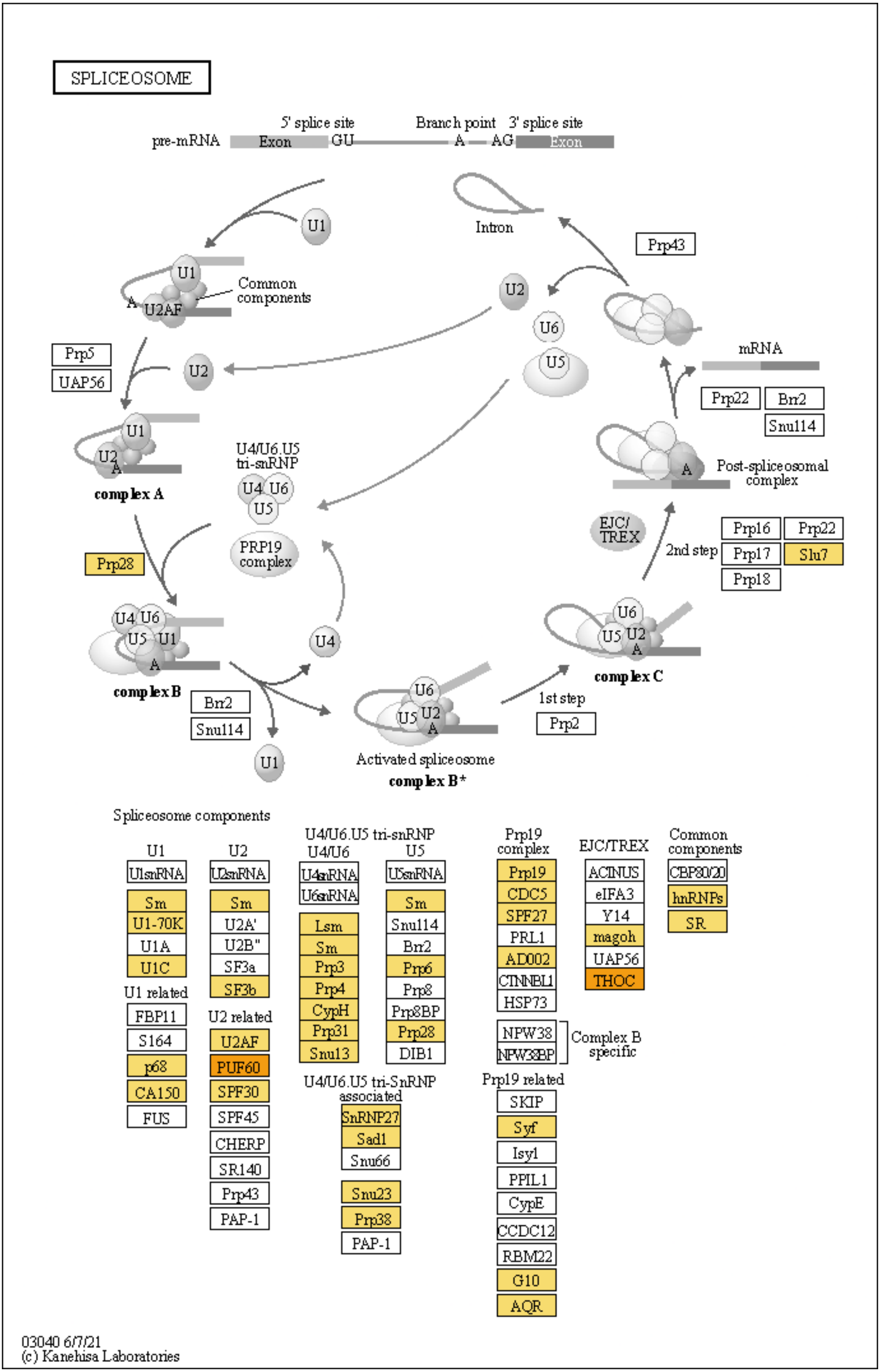
SPLICEOSOME
pre-mRNA
5' splice site
Exon
GU
Branch point
A
3' splice site
AG
Exon
U1
Intron
Prp43
U1
Common components
A
U2AF
U2
U6
U5
Prp5
UAP56
U2
mRNA
U1
U2
A
complex A
U4/U6.U5 tri-snRNP
U4
U6
U5
Prp22
Brr2
Snu114
Post-spliceosomal complex
A
EJC/ TREX
PRP19 complex
Prp28
2nd step
Prp16
Prp17
Prp18
Prp22
Slu7
U4
U6
U5
U1
A
complex B
U4
U6
U5
U2
A
complex C
Brr2
Snu114
U6
U5
U2
A
1st step
Prp2
U1
Activated spliceosome
complex B*
Spliceosome components
U1
U1snRNA
Sm
U1-70K
U1A
U1C
U1 related
FBP11
S164
p68
CA150
FUS
U2
U2snRNA
Sm
U2A'
U2B"
SF3a
SF3b
U2 related
U2AF
PUF60
SPF30
SPF45
CHERP
SR140
Prp43
PAP-1
U4/U6.U5 tri-snRNP
U4/U6
U4snRNA
U6snRNA
Lsm
Sm
Prp3
Prp4
CypH
Prp31
Snu13
U5
U5snRNA
Sm
Snu114
Brr2
Prp6
Prp8
Prp8BP
Prp28
DIB1
U4/U6.U5 tri-SnRNP associated
SnRNP27
Sad1
Snu66
Snu23
Prp38
PAP-1
Prp19 complex
Prp19
CDC5
SPF27
PRL1
AD002
CTNNBL1
HSP73
NPW38
NPW38BP
Complex B specific
Prp19 related
SKIP
Syf
Isy1
PPIL1
CypE
CCDC12
RBM22
G10
AQR
EJC/TREX
ACINUS
eIFA3
Y14
magoh
UAP56
THOC
Common components
CBP80/20
hnRNPs
SR
03040 6/7/21
(c) Kanehisa Laboratories
