# Supplementary material for: Toward novel treatment against filariasis: Insight into genome-wide co-evolutionary analysis of filarial nematodes and Wolbachia
Source: Front Microbiol. 2023 Mar 22;14:1052352. doi: 10.3389/fmicb.2023.1052352 (PMC10073474; doi:10.3389/fmicb.2023.1052352)
Supplement: Supplementary file 1 [file Presentation_1.zip › List of Supplementary Material and Supplementary Figures.pdf]

Supplementary Material

1 Supplementary Data

File S1: Cytoscape session file for network of all top-scoring genes, sub-network of top 5% most highly connected nodes, and sub-networks of chosen GO annotations. Network table for creating Cytoscape session from scratch, and node table with additional details.

File S2: KEGG pathway mapping of prominent pathways

File S3: Cytoscape session file for network of gene, drug targets, and related drugs.Network table for creating Cytoscape session from scratch, and node table with additional details.

2 Supplementary Figures and Tables

2.1 Supplementary Tables

Table S1: GO enrichment of *B. malayi* genes in top-scoring pairs

Table S2: GO enrichment of *Wolbachia* of *B. malayi* genes in top-scoring pairs

Table S3: List of genes in Network 1 (network containing all top 1% most connected nodes and 65% of top 5% most connected nodes)

Table S4: GO enrichment of *B. malayi* genes in Network 1

Table S5: GO enrichment of *Wolbachia* of *B. malayi* genes in Network 1

Table S6: List of top 1% most connected genes and their sequence-matching druggable targets, and associated approved drugs (DrugBank Online results)

2.2 Supplementary Figures

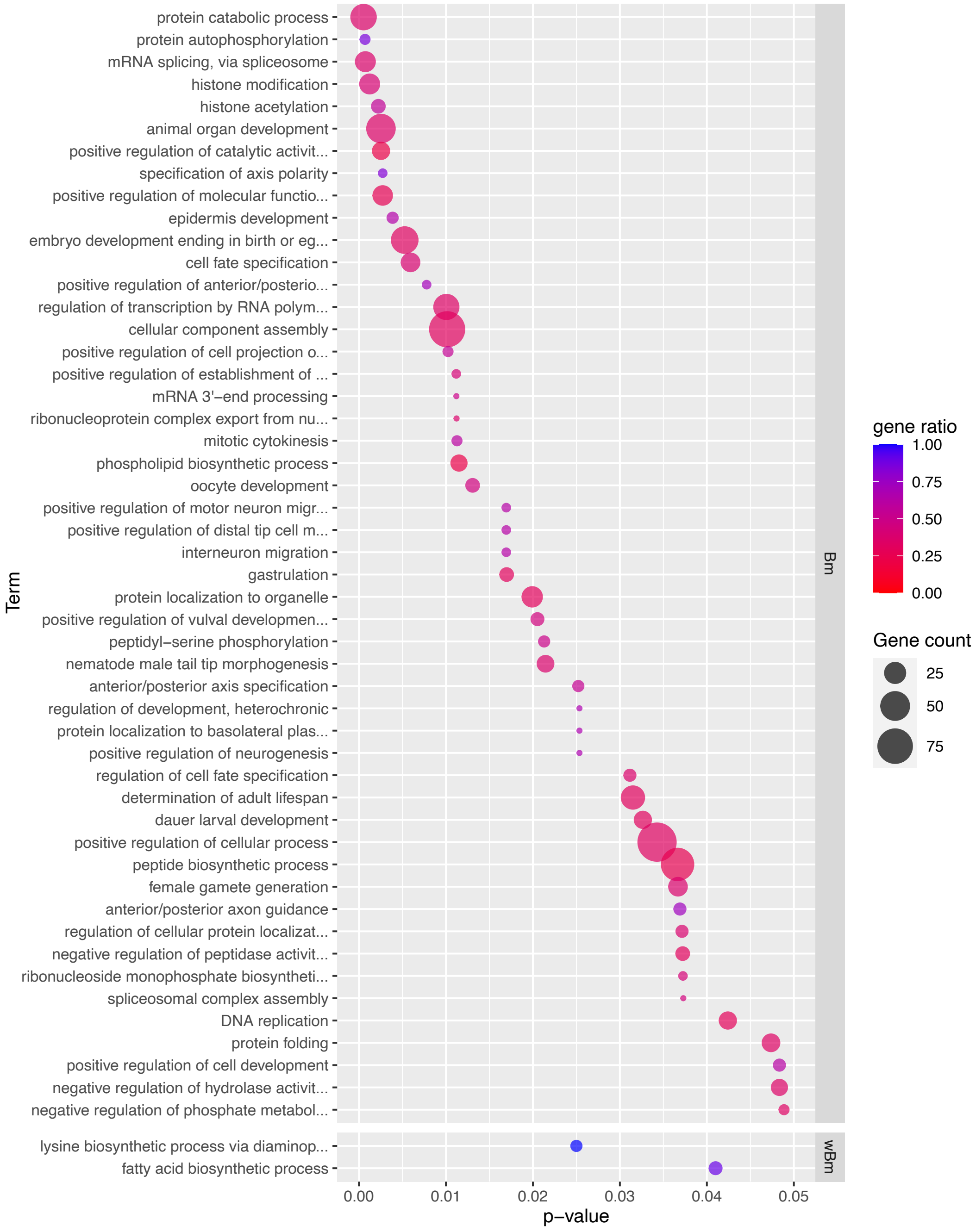

Figure S1: Enriched GO terms of *B. malayi* and *Wolbachia* of *B. malayi* genes in top-scoring pairs (biological process terms only)

All the enriched biological processes (BP) GO terms are shown here, ranked by p-value. P-value referes to the p-value reported by topGO analysis. Gene count indicates the number of genes with that GO term in the input top-scoring gene list. Gene ratio is the number of genes with that GO term in the input list divided by a total number of genes in the genome annotated with that GO term. The complete GO enrichment results with GO ID, full GO term description, and the top-scoring genes in each GO term are in Supplementary Table S1 (for *B. malayi*) and Table S2 (for *Wolbachia* of *B. malayi*).

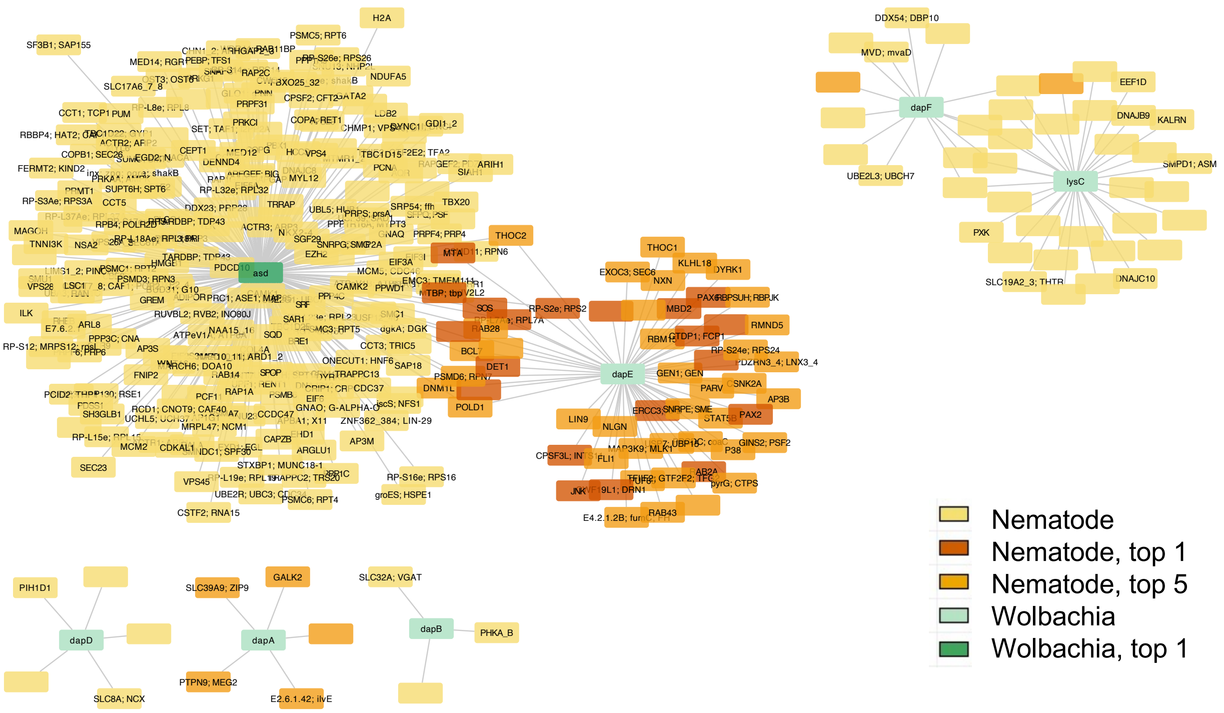

Figure S2: Network of genes co-evolving with *Wolbachia* genes with *lysine synthesis* GO term annotation

The *Wolbachia* genes annotated with GO term *lysine synthesis* (seven nodes in green) and their first neighbours (nematode genes) were selected from the network of all comparisons that passed the cut-off (top-scoring pairs). Colors in yellow-red tone indicate genes in nematodes; blue-green tone indicate genes in *Wolbachia*; with deeper colors indicating the top 5% or top 1% most connected genes. Interactive networks can be found in Cytoscape session file (Supplementary File S1).

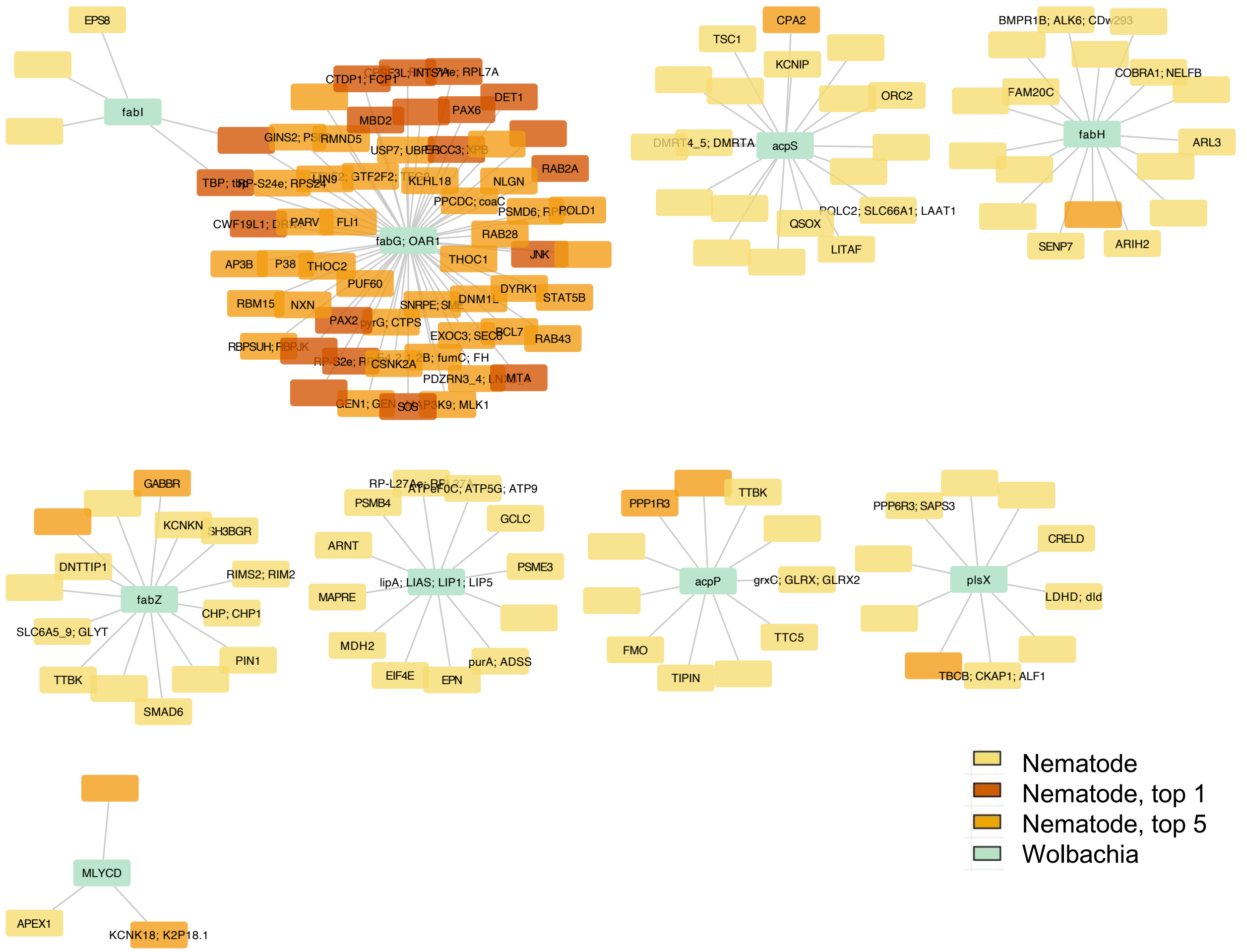

Figure S3: Network of genes co-evolving with *Wolbachia* genes with *fatty acid biosynthesis* GO term annotation

The *Wolbachia* genes annotated with GO term *fatty acid biosynthesis* (nine nodes in green) and their first neighbours (nematode genes) were selected from the network of all comparisons that passed the cut-off (top-scoring pairs). Colors in yellow-red tone indicate genes in nematodes; blue-green tone indicate genes in *Wolbachia*; with deeper colors indicating the top 5% or top 1% most connected genes. Interactive networks can be found in Cytoscape session file (Supplementary File S1).

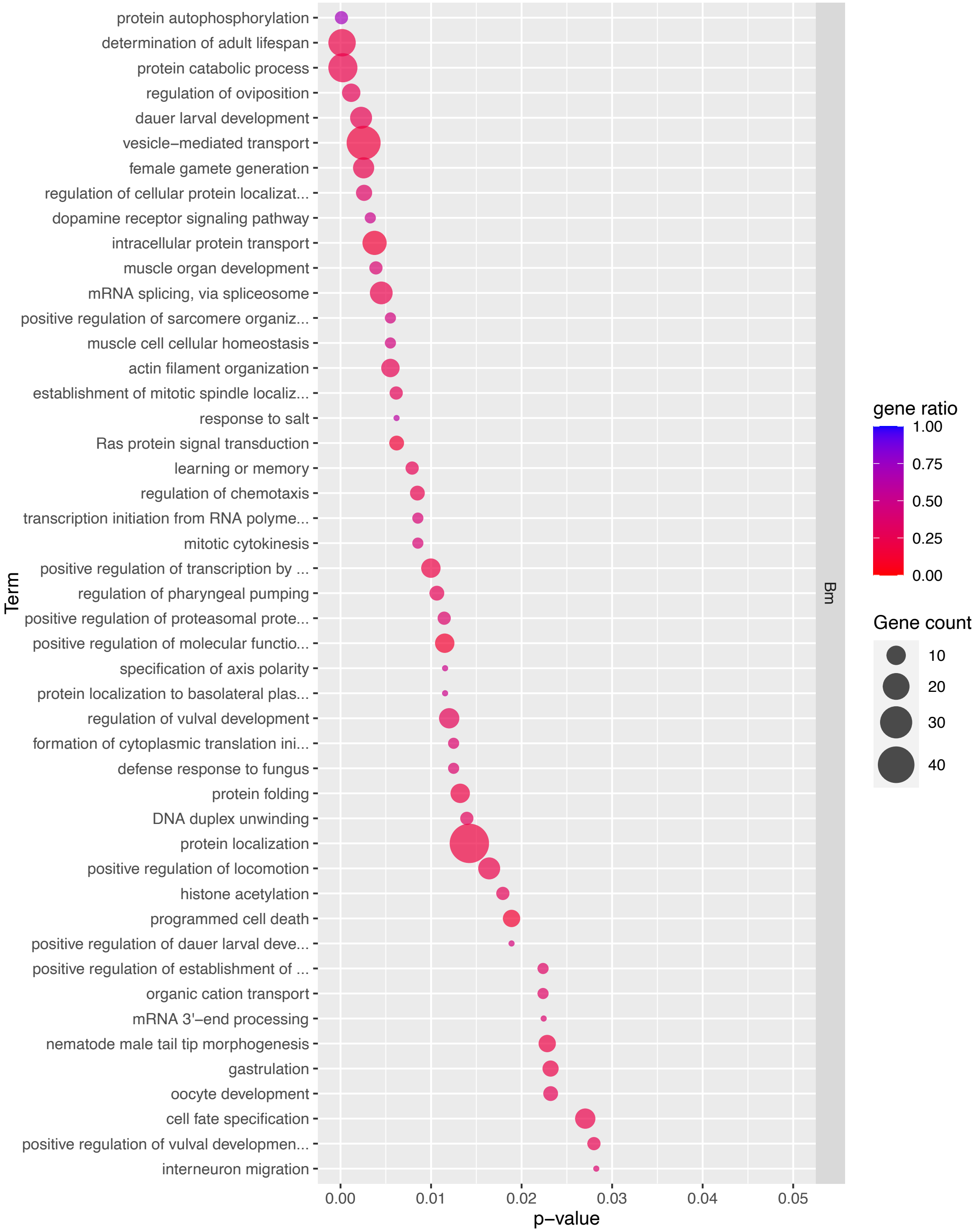

Figure S4: Enriched GO terms *B. malayi* genes in Network 1 (biological process terms only)

All the enriched biological processes (BP) GO terms are shown here, ranked by p-value. P-value referes to the p-value reported by topGO analysis. Gene count indicates the number of genes with that GO term in the input top-scoring gene list. Gene ratio is the number of genes with that GO term in the input list divided by a total number of genes in the genome annotated with that GO term. The complete GO enrichment results with GO ID, full GO term description, and the top-scoring genes in each GO term are in Supplementary Table S4 (for *B. malayi*) and Table S5 (for *Wolbachia* of *B. malayi*).

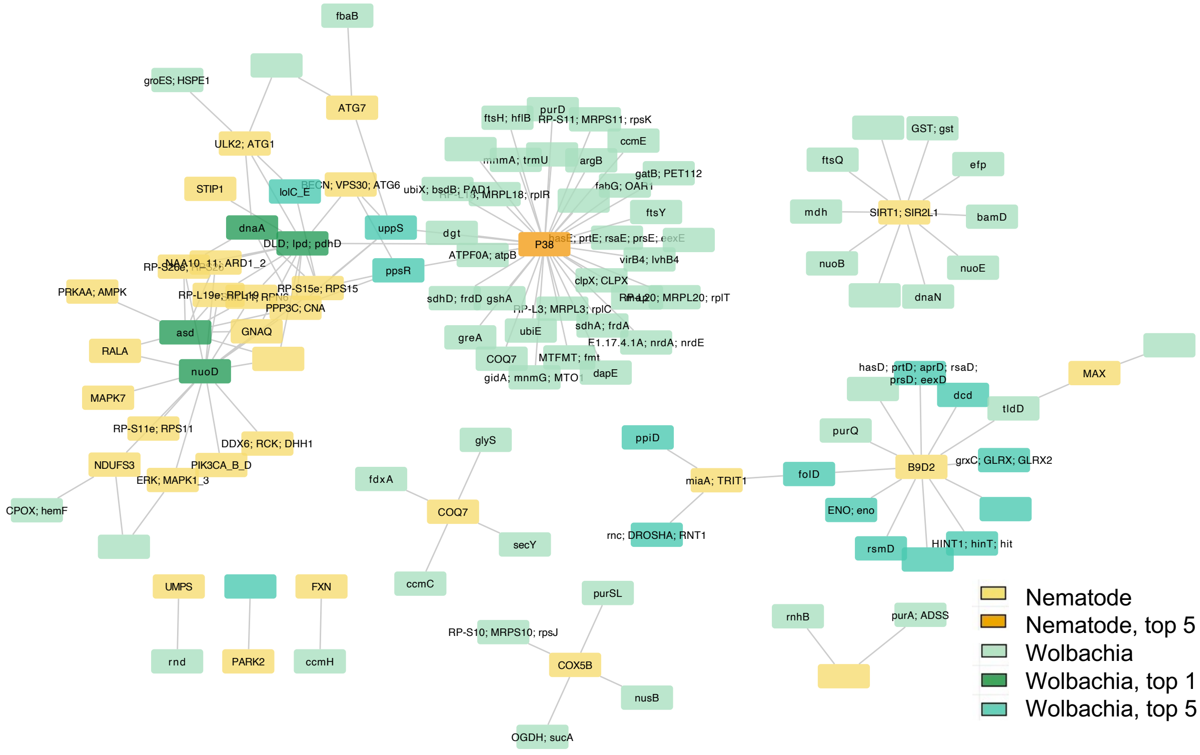

Figure S5: Network of genes co-evolving with *B. malayi* genes with *determination of adult lifespan* GO term annotation

The nematode genes annotated with GO term *determination of adult lifespan* (nodes in yellow and orange) and their first neighbours (*Wolbachia* genes) were selected from all genes that passed the cut-off. Colors in yellow-red tone indicate genes in nematodes; blue-green tone indicate genes in *Wolbachia*; with deeper colors indicating the top 5% or top 1% most connected genes. Interactive networks can be found in Cytoscape session file (Supplementary File S1).
